# Phenotypic and Genotypic Antimicrobial Resistance Profiles of *Flavobacterium psychrophilum* and *Flavobacterium branchiophilum* Isolated From Rainbow Trout ( *Oncorhynchus mykiss* ) in Slovenia

**DOI:** 10.1111/jfd.14119

**Published:** 2025-03-24

**Authors:** Katarina Pavlin, Bojan Papić, Irena Zdovc, Tanja Knific, Igor Gruntar, Rosvita Sitar, Diana Žele Vengušt, Marija Seničar, Matjaž Ocepek, Tanja Švara

**Affiliations:** ^1^ Institute of Pathology, Wild Animals, Fish and Bees, Veterinary Faculty University of Ljubljana Ljubljana Slovenia; ^2^ Institute of Microbiology and Parasitology, Veterinary Faculty University of Ljubljana Ljubljana Slovenia; ^3^ Institute of Food Safety, Feed and Environment, Veterinary Faculty University of Ljubljana Ljubljana Slovenia; ^4^ National Veterinary Institute, Veterinary Faculty University of Ljubljana Ljubljana Slovenia

**Keywords:** AMR profile, *Flavobacterium branchiophilum*, *Flavobacterium psychrophilum*, minimum inhibitory concentration (MIC), rainbow trout ( *Oncorhynchus mykiss* ), whole‐genome sequencing (WGS)

## Abstract

*Flavobacterium psychrophilum* and *Flavobacterium branchiophilum* are important fish pathogens that cause considerable economic losses in freshwater aquaculture worldwide. Their antimicrobial resistance (AMR) profiles were evaluated using the latest Clinical and Laboratory Standards Institute broth microdilution method, which was adjusted for *F. branchiophilum* to achieve growth, and whole‐genome sequencing (WGS). A total of 51 *F. psychrophilum* isolates and eight *F. branchiophilum* isolates from Slovenian farmed rainbow trout underwent phenotypic antimicrobial susceptibility testing. Overall, 86.3% of *F. psychrophilum* isolates were classified as non‐wild‐type (NWT) for oxytetracycline and enrofloxacin, and 90.2% of isolates were NWT for oxolinic acid. In contrast, all isolates tested were classified as wild‐type (WT) for florfenicol and erythromycin. It was not possible to classify *F. branchiophilum* isolates as WT or NWT for the antimicrobials tested. A subset of *F. psychrophilum* (*n* = 7) and *F. branchiophilum* (*n* = 2) isolates was further characterised using WGS to investigate the mechanisms mediating antimicrobial resistance. In *F. psychrophilum* , the T83A or T83V substitution in GyrA was associated with reduced susceptibility to oxolinic acid. No other AMR genes or AMR‐associated mutations were detected.

## 1 Introduction

*Flavobacterium psychrophilum* and *Flavobacterium branchiophilum* are important fish pathogens that severely impact freshwater aquaculture worldwide and lead to considerable economic losses, particularly in the salmonid farming industry (Barnes and Brown 2011; Touchon et al. 2011). *Flavobacterium psychrophilum* is the etiological agent of bacterial cold‐water disease (BCWD) and rainbow trout fry syndrome (RTFS), causing skin and gill lesions and/or systemic disease that can lead to high mortality, especially in juvenile fish (30%–90%) (Barnes and Brown 2011; Davis 1946). *Flavobacterium branchiophilum* is the etiological agent of bacterial gill disease (BGD), has a tropism for the gill epithelium and does not normally invade deeper tissues or internal organs. However, severe infections can lead to high morbidity and mortality (10%–75%) (Davis 1926; Ostland et al. 1995; Speare et al. 1991). Its cultivation is particularly challenging as it is the most fastidious of all known fish pathogenic *Flavobacterium* species (Bernardet and Bowman 2006; Starliper 2011). Therefore, little is known regarding the phenotypic and genotypic diversity of this species (Skulska 2014).

Disease control relies mainly on biosecurity measures and antimicrobial treatment, as no consistently effective commercial vaccine against *F. psychrophilum* or other flavobacteria is yet available (Brenden et al. 2023). Antimicrobial resistance (AMR) in fish pathogens is a growing concern, especially given the limited number of antimicrobials approved for use in aquaculture.

In 2014, the Clinical and Laboratory Standards Institute (CLSI) published the first guideline VET04‐A2 (CLSI 2014) for standardised antimicrobial susceptibility testing (AST) of aquatic bacterial pathogens, including *F. psychrophilum* . To our knowledge, this standard CLSI method has been used in seven studies to date (Jarau et al. 2019; Li et al. 2021; Ngo et al. 2018; Saticioglu et al. 2019; Smith et al. 2016; Söderlund et al. 2018; Van Vliet et al. 2017). In addition, four of these studies used Kronvall's normalised resistance interpretation (NRI) method (Kronvall 2010) to calculate the provisional epidemiological cut‐off values for wild‐type (CO_WT_) and provided their raw MIC data, allowing interpretation and comparison of their results (Ngo et al. 2018; Saticioglu et al. 2019; Smith et al. 2016; Van Vliet et al. 2017). In 2020, the CLSI published the latest standardised guideline VET03 for AST and guideline VET04 presenting the first internationally recognised epidemiological cut‐off values (ECVs) for the assessment of minimum inhibitory concentrations (MICs) for *F. psychrophilum* and *Flavobacterium columnare* (CLSI 2020a, 2020b). Nevertheless, AST and interpretation of results for other *Flavobacterium* species remain challenging.

Studies on the antimicrobial susceptibility of *F. branchiophilum* are scarce. Two studies investigated the susceptibility of *F. branchiophilum* to penicillin G using the disc diffusion method on Anacker and Ordal (AO) agar and concluded that all isolates were susceptible (Ko and Heo 1997; Ostland et al. 1994). Adamek et al. (2018) performed AST using the disc diffusion method and concluded that *F. branchiophilum* isolates were susceptible to florfenicol. However, the lack of standardised guidelines for AST and ECVs for this species limits the validity of the conclusions drawn in these studies.

The mechanisms underlying AMR in *Flavobacterium* spp. are still largely unclear and require further investigation. There are a limited number of studies comparing the genotypic and phenotypic AMR profiles of *F. psychrophilum* (Del Cerro et al. 2010; Henríquez‐Núñez et al. 2012; Izumi and Aranishi 2004; Saticioglu et al. 2019; Shah et al. 2012), and only two studies used whole‐genome sequencing (WGS) to determine the genotypic AMR profile (Park et al. 2021; Söderlund et al. 2018). Several studies investigated the genome characteristics of *F. psychrophilum* (Castillo et al. 2016, 2021; Duchaud et al. 2018; Park et al. 2021; Saticioglu et al. 2024; Söderlund et al. 2018; Wu et al. 2015), while only two such studies are available for *F. branchiophilum* (Kumru et al. 2020; Touchon et al. 2011). WGS provides an alternative approach to predict the AMR profile but requires comprehensive and accurate AMR gene databases, which are currently not available for *Flavobacterium* spp.

The objective of this study was to investigate the phenotypic and genotypic AMR profiles of *F. psychrophilum* and *F. branchiophilum* isolates from Slovenian farmed rainbow trout. For AST, the latest standardised CLSI broth microdilution method for *F. psychrophilum* (CLSI 2020a) was used. We propose an adjustment of this method for *F. branchiophilum* to achieve growth. Genotypic and phenotypic AMR profiles were compared in a subset of *F. psychrophilum* and *F. branchiophilum* isolates. Knowledge of AMR patterns is important to understand the development and transmission of AMR and to develop targeted measures to prevent and control losses caused by pathogenic flavobacteria. To our knowledge, this is the first study to investigate the AMR profiles of *F. branchiophilum* using the broth microdilution method for multiple antimicrobials and the first study to confirm the presence of *F. psychrophilum* and *F. branchiophilum* in Slovenian rainbow trout farms and assess their AMR profiles.

## 2 Materials and Methods

### 2.1 Sampling at Fish Farms

From October 2022 to August 2023, 486 rainbow trout (weight from 3 to 650 g; total length from 6.5 to 40 cm) were sampled from Slovenian fish farms. Longitudinal sampling was conducted at two large fish farms (F1 with spring water source and F2 with river water source) over the four seasons (autumn, winter, spring and summer) from three separate fish tanks. Each sampling included nine diseased fish and nine apparently healthy fish from each tank. Additionally, sampling was carried out in March 2023 at a third fish farm (F3 with river water source) (27 diseased and 27 apparently healthy fish from one fish tank) after the fish farmer reported increased fish mortality. All fish farms use the flow‐through system with concrete tanks.

The diseased fish showed clinical signs such as lethargy, anorexia, swimming abnormalities and opercular flaring, as well as gross changes such as gill anaemia and/or clubbing of the gill filaments, skin and/or fin erosions and ulcerations, skin hyperpigmentation, exophthalmos and splenomegaly. Fish were euthanised individually with an overdose of tricaine methanesulfonate (MS‐222) buffered with sodium bicarbonate (NaHCO_3_) in a 1:2 ratio and immediately necropsied. The dissecting instruments and external surfaces of the fish were disinfected with 70% ethanol before handling the internal organs and after the necropsy of each fish.

### 2.2 Cultivation and Identification of Flavobacteria

Skin and gill samples as well as samples of spleen, kidney and liver were taken with sterile disposable loops and inoculated separately on AO agar plates (Anacker and Ordal 1955), which were incubated at 15°C for 7 d. To obtain pure cultures, subculturing was performed on AO agar at 15°C for 3 d. Isolates were identified using matrix‐assisted laser desorption/ionisation time‐of‐flight mass spectrometry (MALDI‐TOF MS). Single colonies were analysed with the Bruker Microflex LT MS (Bruker Daltonics) using the standard protein extraction method with α‐cyano‐4‐hydroxycinnamic acid as matrix according to the manufacturer's instructions. Mass spectra profiles generated from proteins with masses between 2000 and 20,000 Da were visualised and compared using Bruker flexControl 3.4.207 software and Bruker MBT Compass HT (Version 5.1.550) database. Isolates were stored at −70°C in Microbank cryovials (Pro‐lab Diagnostics, Austin, Tex.) according to the manufacturer's instructions.

Out of 486 rainbow trout sampled, 122 fish tested positive for *F. psychrophilum* and/or *F. branchiophilum* , which were identified with high confidence (log score value above 2.0) using MALDI‐TOF. Specifically, 111 fish were positive for *F. psychrophilum* , seven for *F. branchiophilum* , and four were positive for both pathogens. Most isolates originated from diseased fish; albeit, occasionally, isolates were recovered from apparently healthy fish. Phenotypic AMR profiles were investigated for representative isolates from each positive fish tank at each sampling. Where possible, two isolates from diseased fish and one isolate from apparently healthy fish were selected per positive fish tank. A total of 51 representative *F. psychrophilum* isolates were obtained from gills, skin and/or internal organs (kidney, spleen and liver) of rainbow trout sampled from three fish farms (Table S1). In addition, eight representative *F. branchiophilum* isolates were recovered from the gills of rainbow trout from farms F1 and F2 in the summer of 2023 (Table S1).

### 2.3 Phenotypic AST of *F. psychrophilum*

AST was performed using the broth microdilution method recommended for *F. psychrophilum* in the CLSI guideline VET03 (CLSI 2020a). The MICs for 10 antimicrobials were determined using the Sensititre custom AS1AQ plates (Trek Diagnostic Systems). These were 96‐well dry‐form test plates containing twofold serial dilutions of the following antimicrobials: ampicillin (AMP) 0.015–16 μg/mL (aminopenicillin), ceftazidime (CTZ) 0.03–4 μg/mL (third‐generation cephalosporin), enrofloxacin (ENR) 0.0005–0.25 μg/mL (quinolone), erythromycin (ERY) 0.015–32 μg/mL (macrolide), florfenicol (FLO) 0.03–16 μg/mL (phenicol), gentamicin (GEN) 0.06–8 μg/mL (aminoglycoside), meropenem (MER) 0.008–0.5 μg/mL (carbapenem), oxolinic acid (OXO) 0.002–1 μg/mL (quinolone), oxytetracycline (OXY) 0.015–8 μg/mL (tetracycline) and trimethoprim/sulfamethoxazole (TRS) 0.008/0.15–1/19 μg/mL (potentiated sulphonamide). The layout of the microtitre plate is shown in Figure S2.

The *Escherichia coli* strain ATCC 25922 was used as a quality control strain as recommended in the CLSI guideline VET03 (CLSI 2020a). In addition, the reference strain *F. psychrophilum* ATCC 49418^T^, isolated in 1989, was included for comparison.

A Sensititre autoinoculator (Trek Diagnostic Systems) was used to add the inoculated diluted cation‐adjusted Mueller Hinton broth (DCAMHB) to 96‐well plates. The inoculated plates were covered with plastic adhesive seals, incubated at 18°C and read after 96 h. The ECVs for ENR, ERY, FLO, OXO and OXY against *F. psychrophilum* and the acceptable ranges for the *E. coli* quality control strain are given in CLSI guideline VET04 (CLSI 2020b).

### 2.4 Phenotypic AST of *F. branchiophilum*

The MICs for 10 antimicrobials were determined using the Sensititre custom AS1AQ plates (Trek Diagnostic Systems) as for *F. psychrophilum* . No growth was observed during the initial phenotypic testing of *F. branchiophilum* isolates for an AMR profile according to the CLSI guideline VET03 (CLSI 2020a) recommended for *F. psychrophilum* . Several adjustments of this method were attempted, and growth was achieved by adding 50 mg/L of calcium, magnesium and potassium cations (Skulska 2014) to the sterile distilled water used to dilute the commercial CAMHB (DCAMHB+C). Additionally, better growth was observed when the initial bacterial suspensions were prepared in CAMHB instead of sterile saline and to the upper end of the 0.5 McFarland standard. A Sensititre autoinoculator (Trek Diagnostic Systems) was used to add the inoculated DCAMHB+C to 96‐well plates. To evaluate the growth of *F. branchiophilum* in DCAMHB+C, we also performed the broth microdilution method using the non‐standardised AO broth, which is known to support the growth of this bacterium. The inoculated plates were covered with plastic adhesive seals and incubated at 18°C for 96 h.

The following adjustments were also tested to promote the growth of *F. branchiophilum* but produced no growth: (i) supplementation of full‐strength CAMHB and DCAMHB with 5% fetal bovine serum (Gieseker et al. 2016); (ii) supplementation of full‐strength CAMHB and DCAMHB with 0.11 g of yeast extract (Bernardet and Bowman 2006); and (iii) full‐strength CAMHB (Hesami et al. 2010).

### 2.5 Determination of Wild‐Type (WT) and Non‐Wild‐Type (NWT) Phenotypes

We use the term WT for fully susceptible isolates and NWT for isolates with reduced susceptibility (Silley 2012). For *F. psychrophilum* isolates, WT and NWT populations were defined based on the ECVs for ENR, ERY, FLO, OXO and OXY as provided in the CLSI guideline VET04 (CLSI 2020b). We also used Kronvall's NRI method (Kronvall 2010) to calculate the provisional CO_WT_ values based on the MIC data obtained in this study. The CO_WT_ values were calculated for those antimicrobials for which no more than three of 51 isolates had MICs below or above the tested range and for which the data set generated standard deviations of the normalised distribution lower than 1.2 log_2_ μg/mL. In the calculations, the off‐scale results were assigned MIC values immediately below or above the tested range (Ngo et al. 2018; Saticioglu et al. 2019; Smith et al. 2016). The NRI method was used with the permission of the patent holder, Bioscand AB, TÄBY, Sweden (European patent No. 1383913, US Patent No. 7,465,559). The automatic Excel spreadsheet used to perform the NRI calculations was accessed online (http://www.bioscand.se/nri/) (courtesy of P. Smith, W. Finnegan and G. Kronvall).

Determination of WT and NWT phenotypes for *F. branchiophilum* was not possible due to the lack of a standardised AST method and ECVs for this species. In addition, the NRI method could not be applied due to the low number of isolates tested.

### 2.6 Whole‐Genome Sequencing and Bioinformatics Analysis

To determine the genotypic AMR profiles, a subset of seven *F. psychrophilum* isolates representing different resistotypes based on phenotypic AST and two *F. branchiophilum* isolates from different fish farms were selected for WGS. Six different resistotypes were identified, and one isolate for each was selected for WGS, except for resistotype 6, for which two representative isolates were selected (Table 1). Total DNA was extracted from pure cultures using the DNeasy Blood and Tissue kit (Qiagen, Germantown, MD, USA). DNA libraries were prepared with the Illumina TruSeq DNA Nano Library Prep Kit (Illumina, San Diego, CA, USA). Sequencing was performed on the NextSeq 500 System using 2 × 150 bp chemistry (Illumina, San Diego, CA, USA) to a minimum coverage of 150×. Raw reads were assembled with Shovill version 1.18 (https://github.com/tseemann/shovill) using the—trim option and SPAdes version 3.13.1 (Bankevich et al. 2012) as the underlying assembler with default parameters. AMR genes were identified using ResFinder version 4.1 (Bortolaia et al. 2020) and CARD's Resistance Gene Identifier (RGI) version 6.0.3 (Alcock et al. 2020), both with default parameters. To determine the presence of previously described missense mutations in DNA gyrase subunit A gene (*gyrA*) conferring quinolone resistance, *gyrA* genes were extracted from the assemblies and aligned with the previously published *F. psychrophilum* and *E. coli* *gyrA* genes (Izumi and Aranishi 2004), after which they were translated into amino acid sequences using Geneious Prime version 2022.1.1 (BioMatters Ltd.). Multilocus sequence typing (MLST) was performed using the Sequence Query tool implemented in the PubMLST database for *F. psychrophilum* (Jolley et al. 2018). The molecular serotype of *F. psychrophilum* isolates was predicted based on the presence or absence of three previously described serovar‐associated genes (Rochat et al. 2017), as determined by BLASTn.

### 2.7 Ethics Statement

This study has been approved by the Ethical committee for experiments on animals and the Administration of the Republic of Slovenia for Food Safety, Veterinary Sector and Plant Protection, Ministry of Agriculture, Forestry and Food (approval number: U34401‐10/2022/6).

## 3 Results

### 3.1 Quality Control

The MICs determined for the quality control strain *E. coli* ATCC 25922 were within the acceptable ranges, and the MICs for the reference strain *F. psychrophilum* ATCC 49418^T^ were below the ECVs for *F. psychrophilum* specified in CLSI guideline VET04 (CLSI 2020b). In addition, the reference strain grew at the lower end of the concentration range tested for AMP, GEN and MER, and at the upper end of the concentration range tested for CTZ and TRS. The MICs for the 10 antimicrobials tested against the reference strain *F. psychrophilum* ATCC 49418^T^ are shown in Table S2.

### 3.2 Phenotypic AST Profiles of *F. psychrophilum*

**FIGURE 1:**
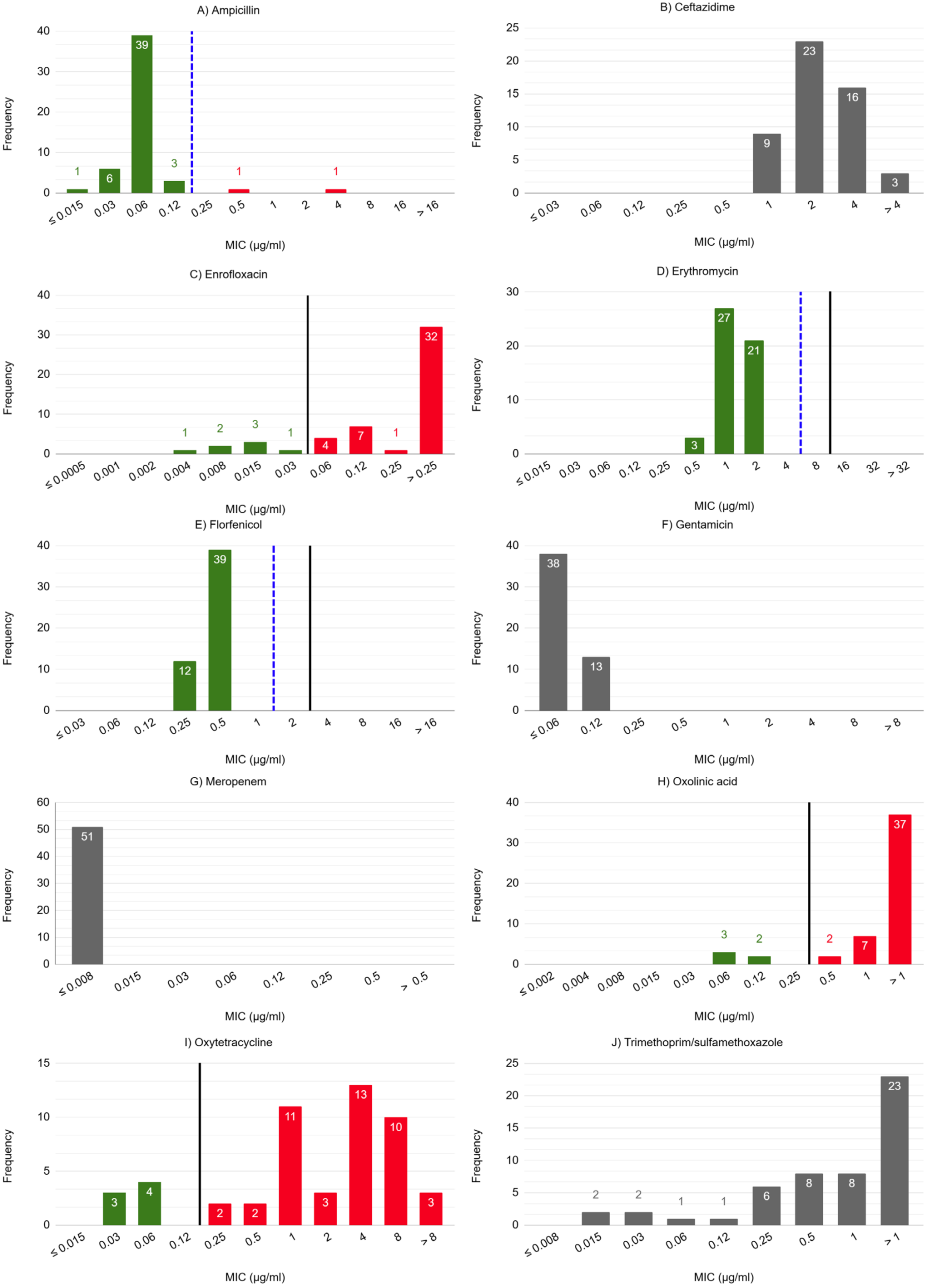
The distribution of minimum inhibitory concentrations (MICs) of 51 *Flavobacterium psychrophilum* isolates for 10 antimicrobials (A–J) determined using the broth microdilution method. The tested concentration range (μg/mL) is presented on the *x*‐axis and the number of isolates on the *y*‐axis. The solid black and dashed blue vertical lines represent the epidemiological cut‐off values (ECVs) and the provisional epidemiological cut‐off values for wild‐type (CO_WT_) for *F. psychrophilum* , respectively. Where possible, the green bars represent the wild‐type (WT) isolates, and the red bars represent the non‐wild‐type (NWT) isolates.

*Flavobacterium psychrophilum* isolates grew well in DCAMHB and formed buttons with smooth or rhizoid edges (Figure S3). The distribution of MICs of 51 *F. psychrophilum* isolates is shown in Figures 1 and S1. The MICs for the individual isolates are shown in Table S2.

For AMP, most isolates exhibited MICs lower than 0.12 μg/mL, while only two isolates were inhibited by higher concentrations, namely 0.5 and 4 μg/mL. Using the provisional CO_WT_ value of ≤ 0.125 μg/mL, 49 isolates (96.1%) were classified as WT and two isolates (3.9%) as NWT (Figure 1A).

The MICs for CTZ were unimodally distributed. All isolates grew at the upper end of the CTZ concentration range tested, with MICs between 1 and > 4 μg/mL. The calculated provisional CO_WT_ value was ≤ 8 μg/mL, which is the first concentration above the tested range for this antimicrobial (Figure 1B).

The MICs for the two quinolones ENR and OXO were bimodally distributed. Using the ECV of ≤ 0.03 μg/mL for ENR, seven isolates (13.7%) were classified as WT and 44 isolates (86.3%) as NWT (Figure 1C). Based on the ECV of ≤ 0.25 μg/mL for OXO, five isolates (9.8%) were classified as WT and 46 isolates (90.2%) as NWT (Figure 1H). In both cases, the most frequently observed MICs exceeded the highest concentration tested for ENR (0.25 μg/mL) and OXO (1 μg/mL). It was not possible to calculate a valid CO_WT_ value for ENR and OXO due to a high percentage of the MICs being ‘above‐scale’.

The MICs for ERY were unimodally distributed. Using the ECV of ≤ 8 μg/mL and the calculated CO_WT_ value of ≤ 4 μg/mL for ERY, all tested isolates were classified as WT (Figure 1D).

The MICs for FLO showed a unimodal distribution. Based on the ECV of ≤ 2 μg/mL and the calculated CO_WT_ value of ≤ 1 μg/mL for FLO, all tested isolates were classified as WT (Figure 1E).

The MICs for GEN were unimodally distributed. All isolates were inhibited by the lower end of the GEN concentration range tested, with MICs between ≤ 0.06 and 0.12 μg/mL. It was not possible to calculate a valid CO_WT_ value due to a high percentage of the MICs being ‘below‐scale’ (Figure 1F).

All isolates were inhibited by the lowest tested concentration of MER (MIC ≤ 0.008 μg/mL). It was not possible to calculate a valid CO_WT_ value due to a high percentage of the MICs being ‘below‐scale’ (Figure 1G).

The distribution of MICs for OXY was bimodal. Using the ECV of ≤ 0.12 μg/mL for OXY, seven isolates (13.7%) were classified as WT and 44 isolates (86.3%) as NWT. It was not possible to calculate a valid CO_WT_ value because the calculated standard deviation for the normalised distribution exceeded < 1.2 log_2_ μg/mL (Figure 1I).

The distribution of MICs for TRS was variable but appeared to be bimodal. Most isolates (88.2%) were inhibited by the upper end of the TRS concentration range tested (MICs ≥ 0.25/4.75 μg/mL), while only six isolates (11.7%) were inhibited by the lower end of the TRS concentration range tested, with MICs between 0.015 and 0.12 μg/mL. The most frequently observed MIC exceeded the highest concentration of TRS (1/19 μg/mL) tested. It was not possible to calculate a valid CO_WT_ value because a high percentage of the MICs were ‘above‐scale’ and the calculated standard deviation for the normalised distribution exceeded < 1.2 log_2_ μg/mL (Figure 1J).

### 3.3 Phenotypic AST Profiles of *F. branchiophilum*

**FIGURE 2:**
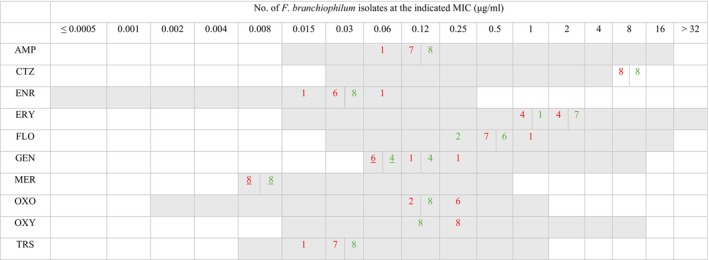
The distribution of minimum inhibitory concentrations (MICs) of eight *Flavobacterium branchiophilum* isolates determined using the broth microdilution method for AMP, ampicillin; CTZ, ceftazidime; ENR, enrofloxacin; ERY, erythromycin; FLO, florfenicol; GEN, gentamicin; MER, meropenem; OXO, oxolinic acid; OXY, oxytetracycline; TRS, trimethoprim/sulfamethoxazole. The range of dilutions tested (μg/mL) for each antimicrobial is shaded grey. MICs higher than the highest concentration tested are given as the first concentration above the tested range, and MICs equal to or lower than the lowest concentration tested are underlined. The red and green numbers represent the MICs determined using diluted cation‐adjusted Mueller Hinton broth with additional cations (DCAMHB+C) and Anacker and Ordal (AO) broth, respectively.

In general, the *F. branchiophilum* isolates grew better in the AO broth than in the DCAMHB+C, as shown by the size of the growth buttons (Figure S4). Nonetheless, the *F. branchiophilum* isolates showed sufficient growth in DCAMHB+C to allow determination of MICs for the tested antimicrobials. Further studies to optimise cation concentration may be required to achieve better growth. Considering the extent of possible technical variation (±1 dilution) in standardised AST, only minor differences were observed between the two media (Figure 2, Table S3). The addition of 5% foetal bovine serum or yeast extract did not improve the growth of *F. branchiophilum* .

Due to the small number of isolates, it was not possible to calculate valid CO_WT_ values and classify the *F. branchiophilum* isolates as either WT or NWT. The MICs for all 10 antimicrobials tested showed a unimodal distribution. The MICs determined by AST using the DCAMHB+C were 0.06–0.12 μg/mL for AMP, > 4 μg/mL for CTZ, 0.015–0.06 μg/mL for ENR, 1–2 μg/mL for ERY, 0.5–1 μg/mL for FLO, ≤ 0.06–0.25 μg/mL for GEN, ≤ 0.008 μg/mL for MER, 0.12–0.25 μg/mL for OXO, 0.25 μg/mL for OXY and 0.015–0.03 μg/mL for TRS. The distribution of MICs of eight *F. branchiophilum* isolates tested using both growth media is shown in Figure 2. The MICs for the individual isolates are shown in Table S3.

### 3.4 Genotypic AMR Profiles of *F. psychrophilum* and *F. branchiophilum* Isolates

Seven *F. psychrophilum* isolates selected for WGS represent six different resistotypes based on phenotypic AST for AMP, ENR, ERY, FLO, OXO and OXY (Table 1). Resistotype 4, which includes isolates that were NWT for ENR, OXO and OXY, was the most prevalent (70.6%). WGS confirmed the presence of a T83A or T83V substitution in GyrA in five of seven sequenced *F. psychrophilum* isolates. All five isolates with this substitution were classified as NWT, and the two isolates without substitution were classified as WT for OXO. The presence of this substitution did not influence the susceptibility of *F. psychrophilum* to ENR (Tables 1 and S2). No AMR genes or other AMR‐associated mutations were detected in the analysed genomes of *F. psychrophilum* and *F. branchiophilum* isolates. Information on the assembly statistics of the reconstructed *Flavobacterium* genomes, including SRA run accession number, total genome size, number of contigs, *N*_50_, GC content, MLST sequence type, MLST allele profile and molecular serotype, is given in Table S4.

**TABLE 1:** Seven *Flavobacterium psychrophilum* isolates selected for whole‐genome sequencing representing six different resistotypes based on phenotypic antimicrobial susceptibility testing for ampicillin (AMP), enrofloxacin (ENR), erythromycin (ERY), florfenicol (FLO), oxolinic acid (OXO) and oxytetracycline (OXY). GyrA substitution associated with resistance to quinolones is also shown.

R	No.	%	Isolate no.	AMP	ENR	ERY	FLO	OXO	OXY	GyrA substitution
**1**	1	2	**9**	0.06	0.015	1	0.25	0.12	0.06	Absent
**2**	2	3.9	**31**	4	> 0.25	1	0.5	**> 1**	1	T83V
**3**	6	11.8	**32**	0.06	0.12	1	0.5	**> 1**	0.06	T83A
**4**	36	70.6	**41**	0.06	> 0.25	1	0.5	**> 1**	8	T83V
**5**	4	7.8	**43**	0.03	0.008	1	0.5	0.06	0.5	Absent
**6**	2	3.9	**38**	0.03	0.03	0.5	0.25	**0.5**	1	T83A
**6**	2	3.9	**50**	≤ 0.015	0.015	1	0.25	**0.5**	0.25	T83A

*Note:* The minimum inhibitory concentrations (MICs; in μg/mL) above the epidemiological cut‐off value (ECV) or the epidemiological cut‐off value for wild‐type (COWT) are marked in red and the MICs below are marked in green. Five of the seven *F. psychrophilum* isolates had MICs for OXO above the ECV and also had a corresponding T83A or T83V substitution in GyrA; these MICs are highlighted in bold and underlined. The remaining two isolates had no such substitution and had MICs for OXO below the ECV.

Abbreviations: %, percentage of isolates exhibiting this resistotype; R, resistotype.

## 4 Discussion

### 4.1 AMR Profiles of *F. psychrophilum*

In this study, antimicrobials approved for use in aquaculture in different countries (Cabello et al. 2013) were tested, including AMP, ENR, ERY, FLO, GEN, OXO, OXY and TRS. CTZ and MER, which are primarily used in human medicine, were also included. FLO and OXY are the only two antimicrobials approved for use in Slovenian aquaculture (JAZMP 2024).

Several studies have used the VET04‐A2 guideline (CLSI 2014) for the AST of *F. psychrophilum* isolates from different countries, including the UK (Ngo et al. 2018; Smith et al. 2016), Sweden (Söderlund et al. 2018), France, Denmark, Finland, Ireland, Chile (Ngo et al. 2018), Turkey (Saticioglu et al. 2019), Canada (Jarau et al. 2019), USA (Ngo et al. 2018; Van Vliet et al. 2017) and China (Li et al. 2021). Most studies reported low NWT frequencies for FLO, low‐to‐moderate NWT frequencies for GEN, β‐lactams (amoxicillin and AMP) and ERY, and moderate‐to‐high NWT frequencies for TRS, OXY and quinolones (OXO, ENR and flumequine). The present results are generally in good agreement with the findings of the studies referenced above, with high MICs for multiple antimicrobial classes. Other referenced studies using the broth microdilution method generated opposing results for some antimicrobials (Henríquez‐Núñez et al. 2012; Hesami et al. 2010). However, it is important to consider that valid comparisons between studies are only possible when standardised and harmonised methods and growth media are used.

Resistant and susceptible *F. psychrophilum* strains with both rhizoid and smooth colony morphotypes are often isolated in individual disease outbreaks. This genotypic and phenotypic heterogeneity poses a challenge for disease control in farm environments (Chen et al. 2008; Del Cerro et al. 2010; Madetoja et al. 2002; Ngo et al. 2017; Sundell et al. 2013). Therefore, it is crucial that the antimicrobial treatment of flavobacterial infections is supported by AST results.

#### 4.1.1 Ampicillin (AMP)

Most isolates (96.1%) were classified as WT and two isolates (3.9%) as NWT for AMP. Several other studies also observed low frequencies (0%–30%) of NWT isolates (Ngo et al. 2018; Saticioglu et al. 2019; Smith et al. 2016; Van Vliet et al. 2017). However, some studies reported high frequencies (71.0%–82.3%) of reduced susceptibility to AMP with MICs exceeding 0.125 μg/mL (Hesami et al. 2010; Li et al. 2021). The calculated provisional CO_WT_ value of ≤ 0.125 μg/mL for AMP is in agreement with some previous studies using the NRI method (Miranda et al. 2016; Ngo et al. 2018; Smith et al. 2016). In addition, Ngo et al. (2018) suggested a high correlation between aminopenicillins (amoxicillin and AMP).

#### 4.1.2 Ceftazidime (CTZ) and Meropenem (MER)

CTZ and MER are primarily used in human medicine to treat severe bacterial infections. CTZ is considered one of the highest priority critically important antimicrobials, and MER is authorised for use in humans only by the World Health Organisation (WHO 2024). To our knowledge, no information is yet available on the susceptibility of *F. psychrophilum* to these antimicrobials. For MER, all isolates displayed MICs below the lowest concentration tested, indicating that they were likely WT strains. In contrast, all isolates grew at the upper end of the CTZ concentration range tested. Given the high MICs observed for CTZ in both the reference *F. psychrophilum* strain and the Slovenian *F. psychrophilum* isolates (Table S2), this indicates potential intrinsic resistance to the antimicrobial. On the other hand, the calculated provisional CO_WT_ value (≤ 8 μg/mL) was higher than the tested range, suggesting that the isolates were WT for CTZ. In view of these results and the unimodal distribution of the MICs, it is not possible to reliably classify the isolates as WT or NWT.

#### 4.1.3 Enrofloxacin (ENR) and Oxolinic Acid (OXO)

Most *F. psychrophilum* isolates were classified as NWT for quinolones, namely 90.2% for OXO and 86.3% for ENR. These results are consistent with several other studies that also report a high frequency (70%–88%) of NWT isolates for quinolones (Henríquez‐Núñez et al. 2012; Hesami et al. 2010; Li et al. 2021; Ngo et al. 2018; Saticioglu et al. 2019; Smith et al. 2016). The high frequency of reduced susceptibility to quinolones is likely due to the widespread use of these antimicrobials in aquaculture, including for the treatment of *F. psychrophilum* infections. Van Vliet et al. (2017) investigated the susceptibility of *F. psychrophilum* isolates from the USA, where the use of quinolones in food animals is restricted, and found no NWT isolates for these agents, supporting this assumption (Saticioglu et al. 2019; Van Vliet et al. 2017). Several studies have found a significant correlation of MIC data for quinolones, suggesting a high level of cross‐resistance in this antimicrobial class. This suggests that OXO may serve as a good representative for quinolones (Ngo et al. 2018; Saticioglu et al. 2019; Smith et al. 2016).

#### 4.1.4 Erythromycin (ERY)

All isolates were classified as WT for ERY, as found in other studies (Jarau et al. 2019; Ngo et al. 2018; Smith et al. 2016; Van Vliet et al. 2017). However, some studies reported low‐to‐moderate frequencies (16.7%–20.0%) of isolates classified as NWT for ERY (Hesami et al. 2010; Saticioglu et al. 2019). ERY is primarily used to treat infections caused by Gram‐positive bacteria and is generally not used to treat *F. psychrophilum* infections. However, in a previous study, it was found to be more effective than FLO in reducing fish mortality and splenic bacterial load (Jarau et al. 2019). Hesami et al. (2010) also stated that ERY could be used to treat *F. psychrophilum* infections and, if used prudently, could delay the emergence of more widespread resistance to FLO.

#### 4.1.5 Florfenicol (FLO)

All isolates were classified as WT for FLO. These results are consistent with the findings in the studies referenced hereafter. In this study, the highest MIC was 0.5 μg/mL, while higher MICs of 1–2 μg/mL were also reported in other studies (Jarau et al. 2019; Li et al. 2021; Ngo et al. 2018; Saticioglu et al. 2019; Smith et al. 2016; Söderlund et al. 2018; Van Vliet et al. 2017). To our knowledge, two NWT isolates (2/31) for FLO (MICs 4 and 8 μg/mL) were found in China (Li et al. 2021), and only two studies reported high frequencies of reduced susceptibility to FLO, namely in 52.8% (38/72) of isolates from Canada (Hesami et al. 2010) and in 92.5% (37/40) of isolates from Chile (Henríquez‐Núñez et al. 2012) with MICs between 4 and 32 μg/mL. In Chile, field reports also indicated a loss of treatment efficacy associated with FLO (Henríquez‐Núñez et al. 2012). FLO has become the new drug of choice for the treatment of *F. psychrophilum* infections in many countries, following the declining efficacy of alternative drugs such as OXY, aminopenicillins and quinolones (Barnes and Brown 2011; Ngo et al. 2018; Saticioglu et al. 2019). Surprisingly, despite the use of FLO in aquaculture for over 30 years, there are few published reports of *F. psychrophilum* strains with reduced susceptibility (Henríquez‐Núñez et al. 2012; Hesami et al. 2010; Li et al. 2021).

#### 4.1.6 Gentamicin (GEN)

All isolates were inhibited by the lower end of the GEN concentration range tested (MICs ≤ 0.06–0.12 μg/mL) and probably represent WT strains. Few studies have investigated the susceptibility of *F. psychrophilum* to GEN, with varying results of low, moderate (≤ 2 μg/mL) (Li et al. 2021; Van Vliet et al. 2017) and high (> 4 μg/mL) (Hesami et al. 2010) MICs. GEN is used in aquaculture against Gram‐negative bacterial infections, but not typically for *F. psychrophilum* (Bojarski et al. 2019).

#### 4.1.7 Oxytetracycline (OXY)

Most isolates (86.3%) showed reduced susceptibility to OXY. These results are in agreement with the findings in other studies, which also report a high frequency (58%–90%) of NWT isolates for OXY (Henríquez‐Núñez et al. 2012; Hesami et al. 2010; Li et al. 2021; Ngo et al. 2018; Saticioglu et al. 2019; Smith et al. 2016). Only one study from the USA reported a low frequency (24%) of isolates classified as NWT for OXY (Van Vliet et al. 2017). OXY is widely used in aquaculture worldwide to treat bacterial infections, including *F. psychrophilum* . Its repeated use is most likely responsible for the occurrence of reduced susceptibility (Saticioglu et al. 2019; Shao 2001). Additionally, according to reports from Slovenian fish veterinarians Rosvita Sitar, MSc, Marija Seničar, MSc, and Dr Diana Žele Vengušt (personal communication, May 20, 2024), the efficacy of this antimicrobial for the treatment of *F. psychrophilum* infections is decreasing.

#### 4.1.8 Trimethoprim/Sulfamethoxazole (TRS)

The distribution of MICs for TRS was variable, ranging from 0.015/0.3 to > 1/19 μg/mL. The most frequently observed MIC was ≥ 1/19 μg/mL, even for the reference *F. psychrophilum* strain. Most isolates (88.2%) were inhibited by the upper end (MICs ≥ 0.25/4.75 μg/mL) of the TRS concentration range tested, as in some studies (Hesami et al. 2010; Ngo et al. 2018), while other studies reported moderate (Saticioglu et al. 2019; Smith et al. 2016) or low (Van Vliet et al. 2017) MICs for TRS. The NRI analysis conducted by Smith et al. (2016) and Ngo et al. (2018) generated high provisional CO_WT_ values, that is, ≤ 1/19 and ≤ 8/152 μg/mL, respectively, leading to the conclusion that their isolates were WT for TRS. It should be noted that, as in this study, all studies that used NRI analysis found that the distribution of MICs for TRS was more scattered than the distributions recorded for other antimicrobials, resulting in standard deviations greater than the limit of < 1.2 log_2_ μg/mL (Ngo et al. 2018; Saticioglu et al. 2019; Smith et al. 2016; Van Vliet et al. 2017). The reason for the large standard deviation remains unclear, making it difficult to establish valid ECVs for the interpretation of MICs. On the other hand, Bruun et al. (2000) suggested that *F. psychrophilum* probably carries intrinsic resistance to potentiated sulphonamides. It was not possible to classify Slovenian *F. psychrophilum* isolates as WT or NWT for TRS.

### 4.2 AMR Profiles of *F. branchiophilum*

The two media used for AST of *F. branchiophilum* gave comparable results and allowed sufficient growth for the determination of MICs. Therefore, DCAMHB+C appears to be a suitable growth medium for AST of *F. branchiophilum* using the broth microdilution method.

There are no established ECVs for this species, and the use of the NRI method was not possible due to the small number of isolates tested in this study; therefore, it was not possible to classify the *F. branchiophilum* isolates as either WT or NWT for any of the antimicrobials. Furthermore, there are no other studies using the broth microdilution method for comparison. There is a significant knowledge gap regarding the antimicrobial resistance of this important fish pathogen, likely due in part to its fastidious nature. We believe that further studies on the AMR of this bacterium using a comparable AST method are urgently needed, regardless of the number of isolates tested, as such results can still provide valuable insights and data for further research, even if only as a preliminary step towards building a more comprehensive knowledge base.

### 4.3 Concordance Between Phenotypic and Genotypic AMR Profiles

Concordance between phenotypic and genotypic AMR profiles was assessed for AMP, ENR, OXO and OXY. However, reliable classification of isolates as WT or NWT for CTZ, GEN, MER and TRS was not possible, and only WT isolates were available for FLO and ERY, which precluded the assessment for these antimicrobials. It should be noted that only a subset of isolates collected between October 2022 and August 2023 was sequenced, limiting the comparison of genotypic and phenotypic AST profiles for all isolates analysed.

The T83A or T83V substitution in GyrA, previously associated with quinolone resistance in *F. psychrophilum* (Izumi and Aranishi 2004; Saticioglu et al. 2019; Shah et al. 2012; Söderlund et al. 2018), was observed in five out of seven sequenced *F. psychrophilum* isolates, which were in perfect concordance with their phenotypic resistance to OXO but not to ENR. However, the genotypic AMR profiling revealed no AMR genes or other AMR‐associated mutations in the studied *F. psychrophilum* and *F. branchiophilum* genomes.

Previous studies have described the presence of AMR genes such as *floR*, *sul2* and various *tet* genes in the *F. psychrophilum* genomes, which have not been associated with phenotypic resistance to FLO, sulphonamides and tetracyclines, respectively (Saticioglu et al. 2019; Söderlund et al. 2018). However, Alvarez et al. (2004) identified the presence of AMR genes such as *cfxA*, *ermF* and *tetQ*, potentially involved in reduced susceptibility to cefoxitin, ERY and tetracycline, respectively. Similar to the present findings, other studies have disproved the association between the presence of various *tet* genes and reduced susceptibility to tetracyclines in *F. psychrophilum* (Saticioglu et al. 2019; Söderlund et al. 2018). In addition, the isolates studied did not have the previously reported six nucleotide polymorphisms in the 16S rRNA gene associated with tetracycline resistance (Soule et al. 2005). Therefore, the genetic background of tetracycline resistance remains unclear.

Verner‐Jeffreys et al. (2017) investigated 377 genomes of the Flavobacteriaceae family (phylum Bacteroidota) and suggested that the transmission of AMR genes commonly associated with Gram‐negative bacteria, particularly from the phylum Pseudomonadota, may be limited or impossible due to genetic incompatibility between these evolutionarily distant bacterial groups. This could mean that the mechanisms leading to reduced susceptibility to antimicrobials are different in flavobacteria than in other bacterial fish pathogens and that the known AMR genes in Pseudomonadota are generally not found in Flavobacteriaceae. Possible barriers to gene transfer and functional expression of AMR genes need to be further investigated (Saticioglu et al. 2019; Verner‐Jeffreys et al. 2017). In addition, AMR genes are often not identified in *Flavobacterium* spp. genomes, and inconsistencies between resistance phenotypes and genotypes have been observed in some studies (Kim et al. 2023; Stine et al. 2017), suggesting the presence of novel AMR genes or complex AMR resistance mechanisms in flavobacteria.

Several studies highlight the importance of efflux pumps, especially the resistance‐nodulation‐division (RND) family and the major facilitator superfamily (MFS), in multidrug resistance of flavobacteria (Castillo et al. 2021; Chen et al. 2017; Chokmangmeepisarn et al. 2021; Clark et al. 2009; Declercq et al. 2021; Mata et al. 2018; Park et al. 2021; Zhang et al. 2017). The importance of efflux pumps in multidrug resistance and pathogenicity has made them a target for the development of new drugs and may represent an interesting new area of research in *Flavobacterium* spp. (Blair et al. 2014; Declercq et al. 2021). Determining AMR patterns and understanding the underlying genetic mechanisms are the first steps towards curbing the further development of AMR.

## 5 Conclusions

This study provides the first assessment of the AMR profiles of *F. psychrophilum* and *F. branchiophilum* isolates in Slovenia. The *F. psychrophilum* isolates were WT for ERY and FLO, while a high frequency of NWT isolates was observed for OXY (86.3%) and quinolones (86.3% for ENR and 90.2% for OXO). A standardised broth microdilution procedure using an appropriate growth medium that supports the growth of *F. branchiophilum* isolates is urgently needed, for which we propose the use of DCAMHB supplemented with calcium, magnesium and potassium cations. Further research comparing the phenotypic and genotypic AMR profiles is needed to identify the genetic mechanisms underlying AMR in *F. psychrophilum* and *F. branchiophilum* .

## Author Contributions

**Katarina Pavlin:** conceptualization, data curation, funding acquisition, investigation, methodology, validation, resources, visualization, writing – original draft. **Bojan Papić:** conceptualization, data curation, formal analysis, investigation, visualization, resources, writing – review and editing. **Irena Zdovc:** conceptualization, investigation, project administration, resources, supervision, writing – review and editing. **Tanja Knific:** conceptualization, data curation, formal analysis, resources, writing – review and editing. **Igor Gruntar:** data curation, resources, writing – review and editing. **Rosvita Sitar:** conceptualization, resources, writing – review and editing. **Diana Žele Vengušt:** conceptualization, resources, writing – review and editing. **Marija Seničar:** conceptualization, resources, writing – review and editing. **Matjaž Ocepek:** conceptualization, supervision, project administration, writing – review and editing. **Tanja Švara:** conceptualization, funding acquisition, project administration, supervision, writing – review and editing.

## Conflicts of Interest

The authors declare no conflicts of interest.

## Supporting information


Data S1.

## Data Availability

The sequencing data have been submitted to the NCBI Sequence Read Archive (SRA) database under BioProject accession number PRJNA1090689. All data are available within this article and its Supporting Information.

## References

[jfd14119-bib-0001] Adamek, M. , F. Teitge , V. Jung‐Schroers , et al. 2018. “Flavobacteria as Secondary Pathogens in Carp Suffering From Koi Sleepy Disease.” Journal of Fish Diseases 41, no. 11: 1631–1642. 10.1111/jfd.12872. 30066956

[jfd14119-bib-0002] Alcock, B. P. , A. R. Raphenya , T. T. Y. Lau , et al. 2020. “CARD 2020: Antibiotic Resistome Surveillance With the Comprehensive Antibiotic Resistance Database.” Nucleic Acids Research 48, no. D1: D517–D525. 10.1093/nar/gkz935. 31665441 PMC7145624

[jfd14119-bib-0003] Alvarez, B. , P. Secades , M. J. McBride , and J. A. Guijarro . 2004. “Development of Genetic Techniques for the Psychrotrophic Fish Pathogen *Flavobacterium psychrophilum* .” Applied and Environmental Microbiology 70, no. 1: 581–587. 10.1128/AEM.70.1.581-587.2004. 14711690 PMC321288

[jfd14119-bib-0004] Anacker, R. L. , and E. J. Ordal . 1955. “Study of a Bacteriophage Infecting the Myxobacterium *Chondrococcus columnaris* .” Journal of Bacteriology 70, no. 6: 738–741. 10.1128/JB.70.6.738-741.1955. 13271323 PMC386281

[jfd14119-bib-0005] Bankevich, A. , S. Nurk , D. Antipov , et al. 2012. “SPAdes: A New Genome Assembly Algorithm and Its Applications to Single‐Cell Sequencing.” Journal of Computational Biology 19, no. 5: 455–477. 10.1089/cmb.2012.0021. 22506599 PMC3342519

[jfd14119-bib-0006] Barnes, M. E. , and M. L. Brown . 2011. “A Review of *Flavobacterium psychrophilum* Biology, Clinical Signs, and Bacterial Cold Water Disease Prevention and Treatment.” Open Fish Science Journal 4, no. 1: 40–48. 10.2174/1874401x01104010040.

[jfd14119-bib-0007] Bernardet, J. F. , and J. P. Bowman . 2006. “The Genus *Flavobacterium* .” In The Prokaryotes: A Handbook on the Biology of bacteria, edited by M. Dworkin , S. Falkow , E. Rosenberg , K. Schleifer , and E. Stackebrandt , vol. 7, 3rd ed., 481–531. Springer Science & Business Media.

[jfd14119-bib-0008] Blair, J. M. A. , G. E. Richmond , and L. J. V. Piddock . 2014. “Multidrug Efflux Pumps in Gram‐Negative bacteria and Their Role in Antibiotic Resistance.” Future Microbiology 9, no. 10: 1165–1177. 10.2217/FMB.14.66. 25405886

[jfd14119-bib-0009] Bojarski, B. , M. Jakubiak , M. Bień , et al. 2019. “Assessment of Gentamicin Effect on Oxidoreductive Balance and Microstructure of Trunk Kidney in Prussian Carp (*Carassius gibelio*).” Annals of Warsaw University of Life Sciences—SGGW, Animal Science 58, no. 2: 115–123. 10.22630/AAS.2019.58.2.12.

[jfd14119-bib-0010] Bortolaia, V. , R. S. Kaas , E. Ruppe , et al. 2020. “ResFinder 4.0 for Predictions of Phenotypes From Genotypes.” Journal of Antimicrobial Chemotherapy 75, no. 12: 3491–3500. 10.1093/jac/dkaa345. 32780112 PMC7662176

[jfd14119-bib-0011] Brenden, T. O. , L. N. Ivan , and T. P. Loch . 2023. “Reducing *Flavobacterium psychrophilum* Transmission Risk via Hatchery‐Rearing Practices: An Individual‐Based Modeling Evaluation.” Aquaculture 563: 738868. 10.1016/j.aquaculture.2022.738868.

[jfd14119-bib-0012] Bruun, M. S. , A. S. Schmidt , L. Madsen , and I. Dalsgaard . 2000. “Antimicrobial Resistance Patterns in Danish Isolates of *Flavobacterium psychrophilum* .” Aquaculture 187, no. 3–4: 201–212. 10.1016/S0044-8486(00)00310-0.

[jfd14119-bib-0013] Cabello, F. C. , H. P. Godfrey , A. Tomova , et al. 2013. “Antimicrobial Use in Aquaculture Re‐Examined: Its Relevance to Antimicrobial Resistance and to Animal and Human Health.” Environmental Microbiology 15, no. 7: 1917–1942. 10.1111/1462-2920.12134. 23711078

[jfd14119-bib-0014] Castillo, D. , R. H. Christiansen , I. Dalsgaard , L. Madsen , R. Espejo , and M. Middelboe . 2016. “Comparative Genome Analysis Provides Insights Into the Pathogenicity of *Flavobacterium psychrophilum* .” PLoS One 11, no. 4: e0152515. 10.1371/journal.pone.0152515. 27071075 PMC4829187

[jfd14119-bib-0015] Castillo, D. , V. L. Donati , J. Jørgensen , et al. 2021. “Comparative Genomic Analyses of *Flavobacterium psychrophilum* Isolates Reveals New Putative Genetic Determinants of Virulence Traits.” Microorganisms 9, no. 8: 1658. 10.3390/microorganisms9081658. 34442736 PMC8400371

[jfd14119-bib-0016] Chen, S. , J. Blom , T. P. Loch , M. Faisal , and E. D. Walker . 2017. “The Emerging Fish Pathogen *Flavobacterium* Spartansii Isolated From Chinook Salmon: Comparative Genome Analysis and Molecular Manipulation.” Frontiers in Microbiology 8: 2339. 10.3389/fmicb.2017.02339. 29250046 PMC5714932

[jfd14119-bib-0017] Chen, Y.‐C. , M. A. Davis , S. E. Lapatra , K. D. Cain , K. R. Snekvik , and D. R. Call . 2008. “Genetic Diversity of *Flavobacterium psychrophilum* Recovered From Commercially Raised Rainbow Trout, *Oncorhynchus mykiss* (Walbaum), and Spawning Coho Salmon, *O. kisutch* (Walbaum).” Journal of Fish Diseases 31, no. 10: 765–773. 10.1111/j.1365-2761.2008.00950.x. 18681900

[jfd14119-bib-0018] Chokmangmeepisarn, P. , P. Thangsunan , P. Kayansamruaj , and C. Rodkhum . 2021. “Resistome Characterization of *Flavobacterium columnare* Isolated From Freshwater Cultured Asian Sea Bass (*Lates calcarifer*) Revealed Diversity of Quinolone Resistance Associated Genes.” Aquaculture 544, no. 1: 737149. 10.1016/j.aquaculture.2021.737149.

[jfd14119-bib-0019] Clark, S. E. , B. A. Jude , G. Russell Danner , and F. A. Fekete . 2009. “Identification of a Multidrug Efflux Pump in *Flavobacterium johnsoniae* .” Veterinary Research 40, no. 6: 55. 10.1051/vetres/2009038. 19558960 PMC2717357

[jfd14119-bib-0020] CLSI . 2014. Methods for Broth Dilution Susceptibility Testing of bacteria Isolated From Aquatic Animals. Approved Guideline VET04‐A2. Clinical and Laboratory Standards Institute.

[jfd14119-bib-0021] CLSI . 2020a. Methods for Antimicrobial Broth Dilution and Disk Diffusion Susceptibility Testing of bacteria Isolated From Aquatic Animals. Approved Guideline VET03. Clinical and Laboratory Standards Institute.

[jfd14119-bib-0022] CLSI . 2020b. Performance Standards for Antimicrobial Susceptibility Testing of bacteria Isolated From Aquatic Animals. Approved Guideline VET04. Clinical and Laboratory Standards Institute.

[jfd14119-bib-0023] Davis, H. S. 1926. “A New Gill Disease of Trout.” Transactions of the American Fisheries Society 56, no. 1: 156–160. 10.1577/1548-8659(1926)56[156:ANGDOT]2.0.CO;2.

[jfd14119-bib-0024] Davis, H. S. 1946. Care and Diseases of Trout. US Fish and Wildlife Service, Research Report 12, 63–66. US Government Printing Office.

[jfd14119-bib-0025] Declercq, A. M. , L. Tilleman , Y. Gansemans , et al. 2021. “Comparative Genomics of *Flavobacterium columnare* Unveils Novel Insights in Virulence and Antimicrobial Resistance Mechanisms.” Veterinary Research 52, no. 1: 1–13. 10.1186/s13567-021-00899-w. 33579339 PMC7881675

[jfd14119-bib-0026] Del Cerro, A. , I. Márquez , and J. M. Prieto . 2010. “Genetic Diversity and Antimicrobial Resistance of *Flavobacterium psychrophilum* Isolated From Cultured Rainbow Trout, *Onchorynchus mykiss* (Walbaum), in Spain.” Journal of Fish Diseases 33, no. 4: 285–291. 10.1111/j.1365-2761.2009.01120.x. 20059636

[jfd14119-bib-0027] Duchaud, E. , T. Rochat , C. Habib , et al. 2018. “Genomic Diversity and Evolution of the Fish Pathogen *Flavobacterium psychrophilum* .” Frontiers in Microbiology 9: 138. 10.3389/fmicb.2018.00138. 29467746 PMC5808330

[jfd14119-bib-0028] Gieseker, C. M. , T. C. Crosby , T. D. Mayer , S. M. Bodeis , and C. B. Stine . 2016. “Development of Similar Broth Microdilution Methods to Determine the Antimicrobial Susceptibility of *Flavobacterium columnare* and *F. psychrophilum* .” Journal of Aquatic Animal Health 28, no. 1: 27–38. 10.1080/08997659.2015.1105878. 26949840 PMC11421677

[jfd14119-bib-0029] Henríquez‐Núñez, H. , O. Evrard , G. Kronvall , and R. Avendaño‐Herrera . 2012. “Antimicrobial Susceptibility and Plasmid Profiles of *Flavobacterium psychrophilum* Strains Isolated in Chile.” Aquaculture 354–355: 38–44. 10.1016/j.aquaculture.2012.04.034.

[jfd14119-bib-0030] Hesami, S. , J. Parkman , J. I. MacInnes , J. T. Gray , C. L. Gyles , and J. S. Lumsden . 2010. “Antimicrobial Susceptibility of *Flavobacterium psychrophilum* Isolates From Ontario.” Journal of Aquatic Animal Health 22, no. 1: 39–49. 10.1577/H09-008.1. 20575364

[jfd14119-bib-0031] Izumi, S. , and F. Aranishi . 2004. “Relationship Between gyrA Mutations and Quinolone Resistance in *Flavobacterium psychrophilum* Isolates.” Applied and Environmental Microbiology 70, no. 7: 3968–3972. 10.1128/AEM.70.7.3968-3972.2004. 15240271 PMC444768

[jfd14119-bib-0032] Jarau, M. , J. I. MacInnes , and J. S. Lumsden . 2019. “Erythromycin and Florfenicol Treatment of Rainbow Trout *Oncorhynchus mykiss* (Walbaum) Experimentally Infected With *Flavobacterium psychrophilum* .” Journal of Fish Diseases 42, no. 3: 325–334. 10.1111/jfd.12944. 30632170

[jfd14119-bib-0033] Jolley, K. A. , J. E. Bray , and M. C. J. Maiden . 2018. “Open‐Access Bacterial Population Genomics: BIGSdb Software, the PubMLST.Org Website and Their Applications [Version 1; Peer Review: 2 Approved].” Wellcome Open Research 3: 124. 10.12688/wellcomeopenres.14826.1. 30345391 PMC6192448

[jfd14119-bib-0034] Kim, Y. S. , E. M. Hwang , C. M. Jeong , and C. J. Cha . 2023. “ *Flavobacterium psychrotrophum* sp. nov. and *Flavobacterium panacagri* sp. nov., Isolated From Freshwater and Soil.” Journal of Microbiology 61, no. 10: 891–901. 10.1007/s12275-023-00081-1. 37851309

[jfd14119-bib-0035] Ko, Y.‐M. , and G.‐J. Heo . 1997. “Characteristics of *Flavobacterium branchiophilum* Isolated From Rainbow Trout in Korea.” Fish Pathology 32, no. 2: 97–102. 10.3147/jsfp.32.97.

[jfd14119-bib-0036] Kronvall, G. 2010. “Normalized Resistance Interpretation as a Tool for Establishing Epidemiological MIC Susceptibility Breakpoints.” Journal of Clinical Microbiology 48, no. 12: 4445–4452. 10.1128/JCM.01101-10. 20926714 PMC3008453

[jfd14119-bib-0037] Kumru, S. , H. C. Tekedar , J. Blom , M. L. Lawrence , and A. Karsi . 2020. “Genomic Diversity in Flavobacterial Pathogens of Aquatic Origin.” Microbial Pathogenesis 142: 104053. 10.1016/j.micpath.2020.104053. 32058022

[jfd14119-bib-0038] Li, S. , J. Chai , C. Knupp , et al. 2021. “Phenotypic and Genetic Characterization of *Flavobacterium psychrophilum* Recovered From Diseased Salmonids in China.” Microbiology Spectrum 9, no. 2: e0033021. 10.1128/spectrum.00330-21. 34523994 PMC8557942

[jfd14119-bib-0039] Madetoja, J. , I. Dalsgaard , and T. Wiklund . 2002. “Occurrence of *Flavobacterium psychrophilum* in Fish‐Farming Environments.” Diseases of Aquatic Organisms 52, no. 2: 109–118. 10.3354/dao052109. 12542087

[jfd14119-bib-0040] Mata, W. , C. Putita , H. T. Dong , P. Kayansamruaj , S. Senapin , and C. Rodkhum . 2018. “Quinolone‐Resistant Phenotype of *Flavobacterium columnare* Isolates Harbouring Point Mutations Both in gyrA and parC but Not in gyrB or parE.” Journal of Global Antimicrobial Resistance 15: 55–60. 10.1016/j.jgar.2018.05.014. 29807204

[jfd14119-bib-0041] Miranda, C. D. , P. Smith , R. Rojas , S. Contreras‐Lynch , and J. M. A. Vega . 2016. “Antimicrobial Susceptibility of *Flavobacterium psychrophilum* From Chilean Salmon Farms and Their Epidemiological Cut‐Off Values Using Agar Dilution and Disk Diffusion Methods.” Frontiers in Microbiology 7: 1880. 10.3389/fmicb.2016.01880. 27933043 PMC5122587

[jfd14119-bib-0042] Ngo, T. P. H. , K. L. Bartie , K. D. Thompson , D. W. Verner‐Jeffreys , R. Hoare , and A. Adams . 2017. “Genetic and Serological Diversity of *Flavobacterium psychrophilum* Isolates From Salmonids in United Kingdom.” Veterinary Microbiology 201: 216–224. 10.1016/j.vetmic.2017.01.032. 28284613

[jfd14119-bib-0043] Ngo, T. P. H. , P. Smith , K. L. Bartie , et al. 2018. “Antimicrobial Susceptibility of *Flavobacterium psychrophilum* Isolates From the United Kingdom.” Journal of Fish Diseases 41, no. 2: 309–320. 10.1111/jfd.12730. 29064104

[jfd14119-bib-0044] Ostland, V. E. , J. S. Lumsden , D. D. Macphee , and H. W. Ferguson . 1994. “Characteristics of *Flavobacterium branchiophilum* , the Cause of Salmonid Bacterial Gill Disease in Ontario.” Journal of Aquatic Animal Health 6, no. 1: 13–26. 10.1577/1548-8667(1994)006<0013:COFBTC>2.3.CO;2.

[jfd14119-bib-0045] Ostland, V. E. , D. D. MacPhee , J. S. Lumsden , and H. W. Ferguson . 1995. “Virulence of *Flavobacterium branchiophilum* in Experimentally Infected Salmonids.” Journal of Fish Diseases 18, no. 3: 249–262.

[jfd14119-bib-0046] Park, J. , H. Roh , Y. Lee , J. Park , and D.‐H. Kim . 2021. “Complete Genome Analysis of *Flavobacterium psychrophilum* Strain FPRT1, Isolated From Diseased Rainbow Trout (*Oncorhynchus mykiss*) in South Korea.” Microbiology Resource Announcements 10, no. 12: e00151‐21. 10.1128/MRA.00151-21. 33766901 PMC7996460

[jfd14119-bib-0047] Rochat, T. , E. Fujiwara‐Nagata , S. Calvez , et al. 2017. “Genomic Characterization of *Flavobacterium psychrophilum* Serotypes and Development of a Multiplex PCR‐Based Serotyping Scheme.” Frontiers in Microbiology 8: 1752. 10.3389/fmicb.2017.01752. 28955320 PMC5601056

[jfd14119-bib-0048] Saticioglu, I. B. , M. Duman , N. Ajmi , S. Altun , T. Rochat , and E. Duchaud . 2024. “Phylogenomic Characterization of *Flavobacterium psychrophilum* Isolates Retrieved From Turkish Rainbow Trout Farms.” Journal of Fish Diseases e13961. 10.1111/jfd.13961. 38773965 PMC12285750

[jfd14119-bib-0049] Saticioglu, I. B. , M. Duman , P. Smith , T. Wiklund , and S. Altun . 2019. “Antimicrobial Resistance and Resistance Genes in *Flavobacterium psychrophilum* Isolates From Turkey.” Aquaculture 512: 734293. 10.1016/j.aquaculture.2019.734293.

[jfd14119-bib-0050] Shah, S. Q. A. , H. Nilsen , K. Bottolfsen , D. J. Colquhoun , and H. Sørum . 2012. “DNA Gyrase and Topoisomerase IV Mutations in Quinolone‐Resistant *Flavobacterium psychrophilum* Isolated From Diseased Salmonids in Norway.” Microbial Drug Resistance 18, no. 2: 207–214. 10.1089/mdr.2011.0142. 22283604

[jfd14119-bib-0051] Shao, Z. J. 2001. “Aquaculture Pharmaceuticals and Biologicals: Current Perspectives and Future Possibilities.” Advanced Drug Delivery Reviews 50, no. 3: 229–243. 10.1016/s0169-409x(01)00159-4. 11500229

[jfd14119-bib-0052] Silley, P. 2012. “Susceptibility Testing Methods, Resistance and Breakpoints: What Do These Terms Really Mean?” Revue Scientifique et Technique 31, no. 1: 33–41. 10.20506/rst.31.1.2097. 22849266

[jfd14119-bib-0053] Skulska, I. 2014. “Culture of the Bacterial Gill Disease Organism, *Flavobacterium branchiophilum* and Strain Differences Relevant to Epizoology.” University of Guelph, Molecular and Cellular Biology. https://api.semanticscholar.org/CorpusID:91169613.

[jfd14119-bib-0054] Smith, P. , R. Endris , G. Kronvall , et al. 2016. “Epidemiological Cut‐Off Values for *Flavobacterium psychrophilum* MIC Data Generated by a Standard Test Protocol.” Journal of Fish Diseases 39, no. 2: 143–154. 10.1111/jfd.12336. 25546427

[jfd14119-bib-0055] Söderlund, R. , M. Hakhverdyan , A. Aspan , and E. Jansson . 2018. “Genome Analysis Provides Insights Into the Epidemiology of Infection With *Flavobacterium psychrophilum* Among Farmed Salmonid Fish in Sweden.” Microbial Genomics 4, no. 12: e000241. 10.1099/mgen.0.000241. 30543323 PMC6412038

[jfd14119-bib-0056] Soule, M. , S. LaFrentz , K. Cain , S. LaPatra , and D. R. Call . 2005. “Polymorphisms in 16S rRNA Genes of *Flavobacterium psychrophilum* Correlate With Elastin Hydrolysis and Tetracycline Resistance.” Diseases of Aquatic Organisms 65, no. 3: 209–216. 10.3354/dao065209. 16119889

[jfd14119-bib-0057] Speare, D. J. , H. W. Ferguson , F. W. M. Beamish , J. A. Yager , and S. Yamashiro . 1991. “Pathology of Bacterial Gill Disease: Sequential Development of Lesions During Natural Outbreaks of Disease.” Journal of Fish Diseases 14, no. 1: 21–32. 10.1111/j.1365-2761.1991.tb00573.x.

[jfd14119-bib-0058] Starliper, C. E. 2011. “Bacterial Coldwater Disease of Fishes Caused by *Flavobacterium psychrophilum* .” Journal of Advanced Research 2, no. 2: 97–108. 10.1016/j.jare.2010.04.001.

[jfd14119-bib-0059] Stine, C. B. , C. Li , T. C. Crosby , N. R. Hasbrouck , C. Lam , and D. A. Tadesse . 2017. “Draft Whole‐Genome Sequences of 18 *Flavobacterium* spp.” Genome Announcements 5, no. 46: e00865‐17. 10.1128/genomeA.00865-17. 29146841 PMC5690318

[jfd14119-bib-0060] Sundell, K. , S. Heinikainen , and T. Wiklund . 2013. “Structure of *Flavobacterium psychrophilum* Populations Infecting Farmed Rainbow Trout *Oncorhynchus mykiss* .” Diseases of Aquatic Organisms 103, no. 2: 111–119. 10.3354/dao02573. 23548361

[jfd14119-bib-0061] Touchon, M. , P. Barbier , J.‐F. Bernardet , et al. 2011. “Complete Genome Sequence of the Fish Pathogen *Flavobacterium branchiophilum* .” Applied and Environmental Microbiology 77, no. 21: 7656–7662. 10.1128/AEM.05625-11. 21926215 PMC3209149

[jfd14119-bib-0062] Van Vliet, D. , T. P. Loch , P. Smith , and M. Faisal . 2017. “Antimicrobial Susceptibilities of *Flavobacterium psychrophilum* Isolates From the Great Lakes Basin, Michigan.” Microbial Drug Resistance 23, no. 6: 791–798. 10.1089/mdr.2016.0103. 28068184

[jfd14119-bib-0063] Verner‐Jeffreys, D. W. , T. Brazier , R. Y. Perez , et al. 2017. “Detection of the Florfenicol Resistance Gene floR in Chryseobacterium Isolates From Rainbow Trout. Exception to the General Rule?” FEMS Microbiology Ecology 93, no. 4: 10. 10.1093/femsec/fix015. 28199699

[jfd14119-bib-0064] Wu, A. K. , A. M. Kropinski , J. S. Lumsden , B. Dixon , and J. I. MacInnes . 2015. “Complete Genome Sequence of the Fish Pathogen *Flavobacterium psychrophilum* ATCC 49418(T.).” Standards in Genomic Sciences 10: 3. 10.1186/1944-3277-10-3. 25685258 PMC4322650

[jfd14119-bib-0065] Zhang, Y. , L. Zhao , W. Chen , et al. 2017. “Complete Genome Sequence Analysis of the Fish Pathogen *Flavobacterium columnare* Provides Insights Into Antibiotic Resistance and Pathogenicity Related Genes.” Microbial Pathogenesis 111: 203–211. 10.1016/j.micpath.2017.08.035. 28867620

[jfd14119-bib-0066] Automatic NRI Calculations for MIC Distributions, for Windows and MAC (2019 Version) . Accessed September 5, 2024. http://www.bioscand.se/nri/.

[jfd14119-bib-0067] Javna agencija Republike Slovenije za zdravila in medicinske pripomočke (JAZMP) [Agency for Medicinal Products and Medical Devices of the Republic of Slovenia] . 2024. “Seznam Zdravil za Uporabo v Veterinarski Medicini [List of Medicinal Products for Use in the Veterinary Medicine].” Accessed September 5, 2024. https://www.jazmp.si/veterinarska‐zdravila/podatki‐o‐zdravilih/seznam‐zdravil/2024‐2/.

[jfd14119-bib-0068] Shovill (1.18 Version) . Accessed July 5, 2023. https://github.com/tseemann/shovill.

[jfd14119-bib-0069] World Health Organization (WHO) . 2024. WHO's List of Medically Important Antimicrobials: A Risk Management Tool for Mitigating Antimicrobial Resistance due to Non‐human Use. World Health Organization. Accessed September 5, 2024. https://cdn.who.int/media/docs/default‐source/gcp/who‐mia‐list‐2024‐lv.pdf?sfvrsn=3320dd3d_2.

