# Supplementary material for: Phenotypic and Genotypic Antimicrobial Resistance Profiles of Flavobacterium psychrophilum and Flavobacterium branchiophilum Isolated From Rainbow Trout ( Oncorhynchus mykiss ) in Slovenia
Source: J Fish Dis. 2025 Mar 24;48(7):e14119. doi: 10.1111/jfd.14119 (PMC12152302; doi:10.1111/jfd.14119)
Supplement: Supplementary file 1 — Data S1. [file JFD-48-e14119-s001.docx]

**SUPPLEMENTARY MATERIAL**

**Phenotypic and genotypic antimicrobial resistance profiles of *Flavobacterium psychrophilum* and *Flavobacterium branchiophilum* isolated from rainbow trout (*Oncorhynchus mykiss*)** **in Slovenia**

**Authors****:** Katarina Pavlin^a,*^, Bojan Papić^b^, Irena Zdovc^b^, Tanja Knific^c^*,* Igor Gruntar^b^, Rosvita Sitar^d^, Diana Žele Vengušt^d^, Marija Seničar^d^, Matjaž Ocepek^b^, Tanja Švara^a^

**Affiliations**

^a^Institute of Pathology, Wild Animals, Fish and Bees, Veterinary Faculty, University of Ljubljana, Gerbičeva ulica 60, 1000 Ljubljana, Slovenia

^b^Institute of Microbiology and Parasitology, Veterinary Faculty, University of Ljubljana, Gerbičeva ulica 60, 1000 Ljubljana, Slovenia

^c^Institute of Food Safety, Feed and Environment, Veterinary Faculty, University of Ljubljana, Gerbičeva ulica 60, 1000 Ljubljana, Slovenia

^d^National Veterinary Institute, Veterinary Faculty, University of Ljubljana, Gerbičeva ulica 60, 1000 Ljubljana, Slovenia

*Corresponding author at: Institute of Pathology, Wild Animals, Fish and Bees, Veterinary Faculty, University of Ljubljana, Gerbičeva ulica 60, 1000 Ljubljana, Slovenia.

*E-mail address:* [katarina.pavlin@vf.uni-lj.si](mailto:katarina.pavlin@vf.uni-lj.si) (K. Pavlin)

**Supplementary Tables**

**Table S1** Information on the 51 *Flavobacterium psychrophilum* and eight *Flavobacterium branchiophilum* isolates from Slovenian rainbow trout (*Oncorhynchus mykiss*), including sampling time, fish farm, fish tank, health status of the fish, source of isolation and other positive organs. Where possible, two representative isolates from diseased fish and one from apparently healthy fish per positive fish tank were selected.

| ISOLATE No. | ISOLATE NAME | *FLAVOBACTERIUM* SPECIES | SAMPLING TIME | FISH FARM, TANK, HEALTH# | ISOLATION  SOURCE | OTHER POSITIVE ORGANS |
| --- | --- | --- | --- | --- | --- | --- |
| **1** | F21 | *F. psychrophilum* | Autumn 2022 | F1T1D1 | kidney | skin, gills |
| **2** | F23 | *F. psychrophilum* | Autumn 2022 | F1T1D2 | skin | - |
| **3** | F27 | *F. psychrophilum* | Autumn 2022 | F1T2D1 | gill | skin, liver |
| **4** | F29 | *F. psychrophilum* | Autumn 2022 | F1T2D2 | kidney | - |
| **5** | F35 | *F. psychrophilum* | Autumn 2022 | F1T3D1 | gill | - |
| **6** | F36 | *F. psychrophilum* | Autumn 2022 | F1T3D2 | kidney | liver |
| **7** | F38 | *F. psychrophilum* | Autumn 2022 | F1T2H1 | skin | - |
| **8** | F82 | *F. psychrophilum* | Autumn 2022 | F2T2D1 | kidney | skin, gill, spleen, liver |
| **9*** | F88 | *F. psychrophilum* | Autumn 2022 | F2T2D2 | skin | - |
| **10** | F202 | *F. psychrophilum* | Winter 2023 | F1T1D1 | spleen | - |
| **11** | F206 | *F. psychrophilum* | Winter 2023 | F1T1D2 | spleen | gill, kidney, liver |
| **12** | F211 | *F. psychrophilum* | Winter 2023 | F1T2D1 | kidney | gill, spleen, liver |
| **13** | F328 | *F. psychrophilum* | Winter 2023 | F1T3D1 | liver | - |
| **14** | F332 | *F. psychrophilum* | Winter 2023 | F1T3D2 | kidney | gill, spleen |
| **15** | F296 | *F. psychrophilum* | Winter 2023 | F1T1H1 | gill | - |
| **16** | F255 | *F. psychrophilum* | Winter 2023 | F1T2H1 | spleen | gill, kidney, liver |
| **17** | F341 | *F. psychrophilum* | Winter 2023 | F1T3H1 | spleen | - |
| **18** | F415 | *F. psychrophilum* | Winter 2023 | F2T1D1 | skin | - |
| **19** | F418 | *F. psychrophilum* | Winter 2023 | F2T2D1 | skin | - |
| **20** | F421 | *F. psychrophilum* | Winter 2023 | F2T2D2 | skin | - |
| **21** | F444 | *F. psychrophilum* | Winter 2023 | F2T3D1 | skin | spleen, liver |
| **22** | F427 | *F. psychrophilum* | Winter 2023 | F2T3D2 | skin | - |
| **23** | F1125 | *F. psychrophilum* | Spring 2023 | F1T1D1 | skin | - |
| **24** | F1126 | *F. psychrophilum* | Spring 2023 | F1T1D2 | skin | - |
| **25** | F937 | *F. psychrophilum* | Spring 2023 | F1T2D1 | skin | kidney |
| **26** | F1127 | *F. psychrophilum* | Spring 2023 | F1T3D1 | skin | - |
| **27** | F1070 | *F. psychrophilum* | Spring 2023 | F1T3D2 | liver | - |
| **28** | F1146 | *F. psychrophilum* | Spring 2023 | F1T2H1 | gill | - |
| **29** | F1147 | *F. psychrophilum* | Spring 2023 | F1T3H1 | skin | - |
| **30** | F1184 | *F. psychrophilum* | Spring 2023 | F2T1D1 | spleen | skin, gill, kidney, liver |
| **31*** | F1186 | *F. psychrophilum* | Spring 2023 | F2T1D2 | spleen | kidney |
| **32*** | F1187 | *F. psychrophilum* | Spring 2023 | F2T2D1 | spleen | skin, kidney, liver |
| **33** | F1188 | *F. psychrophilum* | Spring 2023 | F2T2D1 | spleen | skin, kidney, liver |
| **34** | F1266 | *F. psychrophilum* | Spring 2023 | F2T3D1 | spleen | skin, kidney, liver |
| **35** | F1267 | *F. psychrophilum* | Spring 2023 | F2T3D2 | kidney | spleen, liver |
| **36** | F1268 | *F. psychrophilum* | Spring 2023 | F2T3H1 | liver | - |
| **37** | F1560 | *F. psychrophilum* | Summer 2023 | F1T1D1 | skin | - |
| **38*** | F1516 | *F. psychrophilum* | Summer 2023 | F1T1D2 | skin | - |
| **39** | F1518 | *F. psychrophilum* | Summer 2023 | F1T2D1 | kidney | liver |
| **40** | F1562 | *F. psychrophilum* | Summer 2023 | F1T3D1 | skin | - |
| **41*** | F1519 | *F. psychrophilum* | Summer 2023 | F1T3D2 | liver | skin, gill, spleen, kidney |
| **42** | F1595 | *F. psychrophilum* | Summer 2023 | F1T1H1 | gill | - |
| **43*** | F1719 | *F. psychrophilum* | Summer 2023 | F2T1D1 | liver | skin |
| **44** | F1723 | *F. psychrophilum* | Summer 2023 | F2T1D2 | skin | gill, kidney |
| **45** | F1828 | *F. psychrophilum* | Summer 2023 | F2T2D1 | spleen | skin, kidney, liver |
| **46** | F1819 | *F. psychrophilum* | Summer 2023 | F2T2D2 | gill | skin, spleen, kidney, liver |
| **47** | F1824 | *F. psychrophilum* | Summer 2023 | F2T1H1 | gill | - |
| **48** | F1823 | *F. psychrophilum* | Summer 2023 | F2T2H1 | gill | - |
| **49** | F551 | *F. psychrophilum* | March 2023 | F3D1 | liver | skin, gill, spleen, kidney |
| **50*** | F621 | *F. psychrophilum* | March 2023 | F3D2 | skin | gill |
| **51** | F896 | *F. psychrophilum* | March 2023 | F3H1 | gill | - |
| **52** | F1517 | *F. branchiophilum* | Summer 2023 | F1T2D1 | gill | - |
| **53*** | F1628 | *F. branchiophilum* | Summer 2023 | F1T3D1 | gill | - |
| **54** | F1629 | *F. branchiophilum* | Summer 2023 | F1T3D2 | gill | - |
| **55*** | F1727 | *F. branchiophilum* | Summer 2023 | F2T1D1 | gill | - |
| **56** | F1728 | *F. branchiophilum* | Summer 2023 | F2T2D1 | gill | - |
| **57** | F1862 | *F. branchiophilum* | Summer 2023 | F2T3D1 | gill | - |
| **58** | F1822 | *F. branchiophilum* | Summer 2023 | F2T2H1 | gill | - |
| **59** | F1730 | *F. branchiophilum* | Summer 2023 | F2T3H1 | gill | - |

#F, fish farm; T, fish tank; D, diseased fish; H, apparently healthy fish.

Isolates that were further characterised using whole-genome sequencing (WGS) are marked with an asterisk (*).

**Table S2** Raw data on phenotypic antimicrobial susceptibility testing (minimum inhibitory concentrations in μg/ml) for the 51 Slovenian *Flavobacterium psychrophilum* isolates and the reference strain (Ref) determined using the broth microdilution method for AMP, ampicillin; CTZ, ceftazidime; ENR, enrofloxacin; ERY, erythromycin; FLO, florfenicol; GEN, gentamicin; MER, meropenem; OXO, oxolinic acid; OXY, oxytetracycline; TRS, trimethoprim/sulfamethoxazole.

| **No.** | **AMP** | **CTZ** | **ENR** | **ERY** | **FLO** | **GEN** | **MER** | **OXO** | **OXY** | **TRS** |
| --- | --- | --- | --- | --- | --- | --- | --- | --- | --- | --- |
| **Ref** | 0.12 | 2 | 0.015 | 1 | 0.5 | 0.25 | ≤ 0.008 | 0.12 | 0.06 | > 1/19 |
| **1** | 0.06 | 4 | > 0.25 | 2 | 0.5 | ≤ 0.06 | ≤ 0.008 | > 1 | 8 | 0.5/9.5 |
| **2** | 0.06 | 4 | 0.12 | 1 | 0.5 | 0.12 | ≤ 0.008 | > 1 | > 8 | 0.25/4.75 |
| **3** | 0.06 | 4 | > 0.25 | 1 | 0.5 | ≤ 0.06 | ≤ 0.008 | > 1 | 4 | 1/19 |
| **4** | 0.06 | 4 | 0.25 | 1 | 0.5 | 0.12 | ≤ 0.008 | 1 | > 8 | 1/19 |
| **5** | 0.06 | 2 | > 0.25 | 1 | 0.25 | ≤ 0.06 | ≤ 0.008 | > 1 | 4 | 0.25/4.75 |
| **6** | 0.06 | 1 | > 0.25 | 1 | 0.5 | ≤ 0.06 | ≤ 0.008 | > 1 | 4 | > 1/19 |
| **7** | 0.06 | 4 | > 0.25 | 2 | 0.5 | ≤ 0.06 | ≤ 0.008 | > 1 | 4 | 0.5/9.5 |
| **8** | 0.06 | 2 | > 0.25 | 1 | 0.5 | ≤ 0.06 | ≤ 0.008 | > 1 | 8 | > 1/19 |
| **9*** | 0.06 | 4 | 0.015 | 1 | 0.25 | ≤ 0.06 | ≤ 0.008 | 0.12 | 0.06 | > 1/19 |
| **10** | 0.06 | 2 | 0.12 | 1 | 0.25 | ≤ 0.06 | ≤ 0.008 | 1 | 1 | 1/19 |
| **11** | 0.06 | 2 | > 0.25 | 2 | 0.5 | ≤ 0.06 | ≤ 0.008 | > 1 | 8 | > 1/19 |
| **12** | 0.06 | 2 | 0.12 | 1 | 0.5 | 0.12 | ≤ 0.008 | > 1 | 1 | > 1/19 |
| **13** | 0.06 | 4 | > 0.25 | 1 | 0.5 | ≤ 0.06 | ≤ 0.008 | 1 | 4 | 0.5/9.5 |
| **14** | 0.06 | 4 | > 0.25 | 2 | 0.5 | ≤ 0.06 | ≤ 0.008 | > 1 | 4 | 0.5/9.5 |
| **15** | 0.06 | 4 | > 0.25 | 2 | 0.5 | ≤ 0.06 | ≤ 0.008 | > 1 | 4 | 0.25/4.75 |
| **16** | 0.06 | 2 | 0.12 | 1 | 0.25 | ≤ 0.06 | ≤ 0.008 | > 1 | 1 | > 1/19 |
| **17** | 0.06 | 2 | > 0.25 | 2 | 0.5 | ≤ 0.06 | ≤ 0.008 | > 1 | 8 | > 1/19 |
| **18** | 0.06 | 2 | > 0.25 | 2 | 0.5 | ≤ 0.06 | ≤ 0.008 | > 1 | 8 | > 1/19 |
| **19** | 0.03 | 4 | 0.06 | 1 | 0.25 | ≤ 0.06 | ≤ 0.008 | 1 | 4 | > 1/19 |
| **20** | 0.06 | 2 | > 0.25 | 1 | 0.5 | ≤ 0.06 | ≤ 0.008 | > 1 | 4 | 1/19 |
| **21** | 0.03 | 2 | > 0.25 | 1 | 0.25 | ≤ 0.06 | ≤ 0.008 | > 1 | 0.03 | 0.12/2.38 |
| **22** | 0.5 | 4 | 0.06 | 1 | 0.5 | 0.12 | ≤ 0.008 | 1 | 4 | 0.25/4.75 |
| **23** | 0.06 | 2 | > 0.25 | 2 | 0.5 | ≤ 0.06 | ≤ 0.008 | > 1 | 2 | > 1/19 |
| **24** | 0.06 | 4 | > 0.25 | 2 | 0.5 | 0.12 | ≤ 0.008 | > 1 | 1 | 1/19 |
| **25** | 0.06 | 1 | > 0.25 | 1 | 0.5 | ≤ 0.06 | ≤ 0.008 | > 1 | 0.03 | 0.5/9.5 |
| **26** | 0.03 | 4 | 0.06 | 1 | 0.5 | ≤ 0.06 | ≤ 0.008 | 1 | 2 | 0.25/4.75 |
| **27** | 0.06 | 1 | > 0.25 | 1 | 0.5 | ≤ 0.06 | ≤ 0.008 | > 1 | > 8 | > 1/19 |
| **28** | 0.06 | 2 | 0.06 | 0.5 | 0.5 | ≤ 0.06 | ≤ 0.008 | 1 | 2 | 0.5/9.5 |
| **29** | 0.06 | > 4 | > 0.25 | 2 | 0.5 | 0.12 | ≤ 0.008 | > 1 | 1 | 1/19 |
| **30** | 0.06 | 2 | > 0.25 | 2 | 0.5 | 0.12 | ≤ 0.008 | > 1 | 1 | > 1/19 |
| **31*** | 4 | 1 | > 0.25 | 1 | 0.5 | ≤ 0.06 | ≤ 0.008 | **> 1** | 1 | 0.5/9.5 |
| **32*** | 0.06 | 2 | 0.12 | 1 | 0.5 | ≤ 0.06 | ≤ 0.008 | **> 1** | 0.06 | > 1/19 |
| **33** | 0.06 | 2 | 0.12 | 2 | 0.5 | 0.12 | ≤ 0.008 | > 1 | 0.03 | > 1/19 |
| **34** | 0.06 | 2 | > 0.25 | 2 | 0.5 | ≤ 0.06 | ≤ 0.008 | > 1 | 8 | > 1/19 |
| **35** | 0.06 | 1 | > 0.25 | 1 | 0.25 | ≤ 0.06 | ≤ 0.008 | > 1 | 8 | > 1/19 |
| **36** | 0.03 | 2 | 0.004 | 1 | 0.5 | ≤ 0.06 | ≤ 0.008 | 0.06 | 0.25 | 0.03/0.6 |
| **37** | 0.06 | 2 | 0.008 | 0.5 | 0.25 | 0.12 | ≤ 0.008 | 0.06 | 0.5 | 0.03/0.6 |
| **38*** | 0.03 | 2 | 0.03 | 0.5 | 0.25 | ≤ 0.06 | ≤ 0.008 | **0.5** | 1 | 0.015/0.3 |
| **39** | 0.06 | 4 | > 0.25 | 1 | 0.5 | ≤ 0.06 | ≤ 0.008 | > 1 | 4 | 0.25/4.75 |
| **40** | 0.06 | 4 | > 0.25 | 2 | 0.5 | 0.12 | ≤ 0.008 | > 1 | 1 | 1/19 |
| **41*** | 0.06 | 1 | > 0.25 | 1 | 0.5 | ≤ 0.06 | ≤ 0.008 | **> 1** | 8 | > 1/19 |
| **42** | 0.12 | > 4 | > 0.25 | 2 | 0.5 | ≤ 0.06 | ≤ 0.008 | > 1 | 4 | 0.5/9.5 |
| **43*** | 0.03 | 1 | 0.008 | 1 | 0.5 | ≤ 0.06 | ≤ 0.008 | 0.06 | 0.5 | 0.015/0.3 |
| **44** | 0.12 | > 4 | 0.015 | 2 | 0.5 | ≤ 0.06 | ≤ 0.008 | 0.12 | 4 | > 1/19 |
| **45** | 0.06 | 1 | > 0.25 | 2 | 0.5 | 0.12 | ≤ 0.008 | > 1 | 8 | > 1/19 |
| **46** | 0.06 | 2 | > 0.25 | 2 | 0.25 | ≤ 0.06 | ≤ 0.008 | > 1 | 1 | > 1/19 |
| **47** | 0.06 | 2 | > 0.25 | 1 | 0.5 | ≤ 0.06 | ≤ 0.008 | > 1 | 8 | 1/19 |
| **48** | 0.06 | 2 | > 0.25 | 2 | 0.5 | 0.12 | ≤ 0.008 | > 1 | 0.06 | > 1/19 |
| **49** | 0.06 | 2 | 0.12 | 2 | 0.25 | 0.12 | ≤ 0.008 | > 1 | 1 | > 1/19 |
| **50*** | ≤ 0.015 | 1 | 0.015 | 1 | 0.25 | ≤ 0.06 | ≤ 0.008 | **0.5** | 0.25 | 0.06/1.19 |
| **51** | 0.12 | 4 | > 0.25 | 2 | 0.5 | ≤ 0.06 | ≤ 0.008 | > 1 | 0.06 | > 1/19 |

Seven isolates that were further characterised using whole-genome sequencing are marked with an asterisk (*). Five of the seven *Flavobacterium psychrophilum* isolates had MICs for OXO above the epidemiological cut-off value (ECV) and also had a corresponding T83A or T83V substitution in GyrA; these MICs are highlighted in bold and underlined. The remaining two isolates had no such substitution and had MICs for OXO below the ECV. For antimicrobials for which the ECV is defined in the CLSI guideline VET04 (CLSI 2020) or for which a provisional epidemiological cut-off value for wild type (CO_WT_) could be calculated, the MICs above the ECV or CO_WT_ are marked in red and the MICs below are marked in green.

**Table S3** Raw data on phenotypic antimicrobial susceptibility testing (minimum inhibitory concentrations in μg/ml) for the eight Slovenian *Flavobacterium branchiophilum* isolates determined using the broth microdilution method for AMP, ampicillin; CTZ, ceftazidime; ENR, enrofloxacin; ERY, erythromycin; FLO, florfenicol; GEN, gentamicin; MER, meropenem; OXO, oxolinic acid; OXY, oxytetracycline; TRS, trimethoprim/sulfamethoxazole.

|  | **AMP** | | **CTZ** | | **ENR** | | **ERY** | | **FLO** | | **GEN** | | **MER** | | **OXO** | | **OXY** | | **TRS** | |
| --- | --- | --- | --- | --- | --- | --- | --- | --- | --- | --- | --- | --- | --- | --- | --- | --- | --- | --- | --- | --- |
| **No.** | A | M | A | M | A | M | A | M | A | M | A | M | A | M | A | M | A | M | A | M |
| **52** | 0.12 | 0.12 | > 4 | > 4 | 0.03 | 0.06 | 2 | 1 | 0.5 | 0.5 | 0.12 | ≤ 0.06 | ≤ 0.008 | ≤ 0.008 | 0.12 | 0.25 | 0.12 | 0.25 | 0.03/0.6 | 0.03/0.6 |
| **53*** | 0.12 | 0.12 | > 4 | > 4 | 0.03 | 0.015 | 2 | 1 | 0.5 | 0.5 | ≤ 0.06 | 0.12 | ≤ 0.008 | ≤ 0.008 | 0.12 | 0.12 | 0.12 | 0.25 | 0.03/0.6 | 0.03/0.6 |
| **54** | 0.12 | 0.12 | > 4 | > 4 | 0.03 | 0.03 | 2 | 2 | 0.5 | 0.5 | ≤ 0.06 | ≤ 0.06 | ≤ 0.008 | ≤ 0.008 | 0.12 | 0.25 | 0.12 | 0.25 | 0.03/0.6 | 0.03/0.6 |
| **55*** | 0.12 | 0.06 | > 4 | > 4 | 0.03 | 0.03 | 2 | 1 | 0.5 | 0.5 | 0.12 | ≤ 0.06 | ≤ 0.008 | ≤ 0.008 | 0.12 | 0.25 | 0.12 | 0.25 | 0.03/0.6 | 0.03/0.6 |
| **56** | 0.12 | 0.12 | > 4 | > 4 | 0.03 | 0.03 | 1 | 2 | 0.25 | 0.5 | ≤ 0.06 | ≤ 0.06 | ≤ 0.008 | ≤ 0.008 | 0.12 | 0.25 | 0.12 | 0.25 | 0.03/0.6 | 0.03/0.6 |
| **57** | 0.12 | 0.12 | > 4 | > 4 | 0.03 | 0.03 | 2 | 2 | 0.5 | 1 | 0.12 | 0.25 | ≤ 0.008 | ≤ 0.008 | 0.12 | 0.25 | 0.12 | 0.25 | 0.03/0.6 | 0.03/0.6 |
| **58** | 0.12 | 0.12 | > 4 | > 4 | 0.03 | 0.03 | 2 | 2 | 0.25 | 0.5 | ≤ 0.06 | ≤ 0.06 | ≤ 0.008 | ≤ 0.008 | 0.12 | 0.12 | 0.12 | 0.25 | 0.03/0.6 | 0.015/0.3 |
| **59** | 0.12 | 0.12 | > 4 | > 4 | 0.03 | 0.03 | 2 | 1 | 0.5 | 0.5 | 0.12 | ≤ 0.06 | ≤ 0.008 | ≤ 0.008 | 0.12 | 0.25 | 0.12 | 0.25 | 0.03/0.6 | 0.03/0.6 |

Two isolates that were further characterised using whole-genome sequencing are marked with an asterisk (*). None of the isolates had a T83V or T83A substitution in GyrA. The growth media used for each antimicrobial are specified in the second row with the abbreviations A for Anacker and Ordal (AO) broth and M for diluted cation-adjusted Mueller Hinton broth supplemented with additional cations (DCAMHB+C).

**Table S4** Whole-genome sequencing data on seven *Flavobacterium psychrophilum* and two *Flavobacterium branchiophilum* isolates, including SRA run accession number, total genome size, number of contigs, *N*_50_, GC content, sequence type based on multilocus sequence typing (MLST ST), MLST allele profile, and molecular serotype.

| **No.** | **Isolate name** | ***Flavobacterium* species** | **SRA run accession number** | **Assembly size [Mbp]** | **No. of contigs (>500 bp)** | ***N*_50_ [bp]** | **GC content [%]** | **MLST ST** | **MLST allele profile** | **Molecular serotype** |
| --- | --- | --- | --- | --- | --- | --- | --- | --- | --- | --- |
| 9 | F88 | *F. psychrophilum* | SRR28415848 | 2.76 | 73 | 157870 | 32.54 | ST385 (new) | 1, 2, 1, 1, 1, 1, 2 | Type-2 |
| 31 | F1186 | *F. psychrophilum* | SRR28415853 | 2.74 | 75 | 133336 | 32.35 | ST3 (CC-ST10) | 2, 2, 2, 2, 2, 3, 2 | Type-0 |
| 32 | F1187 | *F. psychrophilum* | SRR28415847 | 2.7 | 76 | 131395 | 32.48 | ST108 (CC-ST01) | 1, 1, 2, 1, 1, 1, 41 | Type-3 |
| 38 | F1516 | *F. psychrophilum* | SRR28415850 | 2.98 | 142 | 110910 | 32.29 | ST401 (new) | 3, 2, 3, 103, 2, 4, 3 | Type-0 |
| 41 | F1519 | *F. psychrophilum* | SRR28415849 | 2.76 | 83 | 172390 | 32.32 | ST92 (CC-ST10) | 2, 2, 2, 2, 2, 3, 41 | Type-2 |
| 43 | F1719 | *F. psychrophilum* | SRR28415852 | 2.99 | 153 | 79657 | 32.32 | ST402 (new) | 3, 22, 3, 43, 2, 3, 3 | Type-0 |
| 50 | F621 | *F. psychrophilum* | SRR28415851 | 3.02 | 165 | 74217 | 32.33 | ST400 (new) | 3, 22, 3, 86, 3, 2, 3 | Type-0 |
| 53 | F1628 | *F. branchiophilum* | SRR28415854 | 3.58 | 82 | 136724 | 32.75 | NA | NA | NA |
| 55 | F1727 | *F. branchiophilum* | SRR28415855 | 3.59 | 81 | 128306 | 32.74 | NA | NA | NA |

NA, not available

**Supplementary Figures**


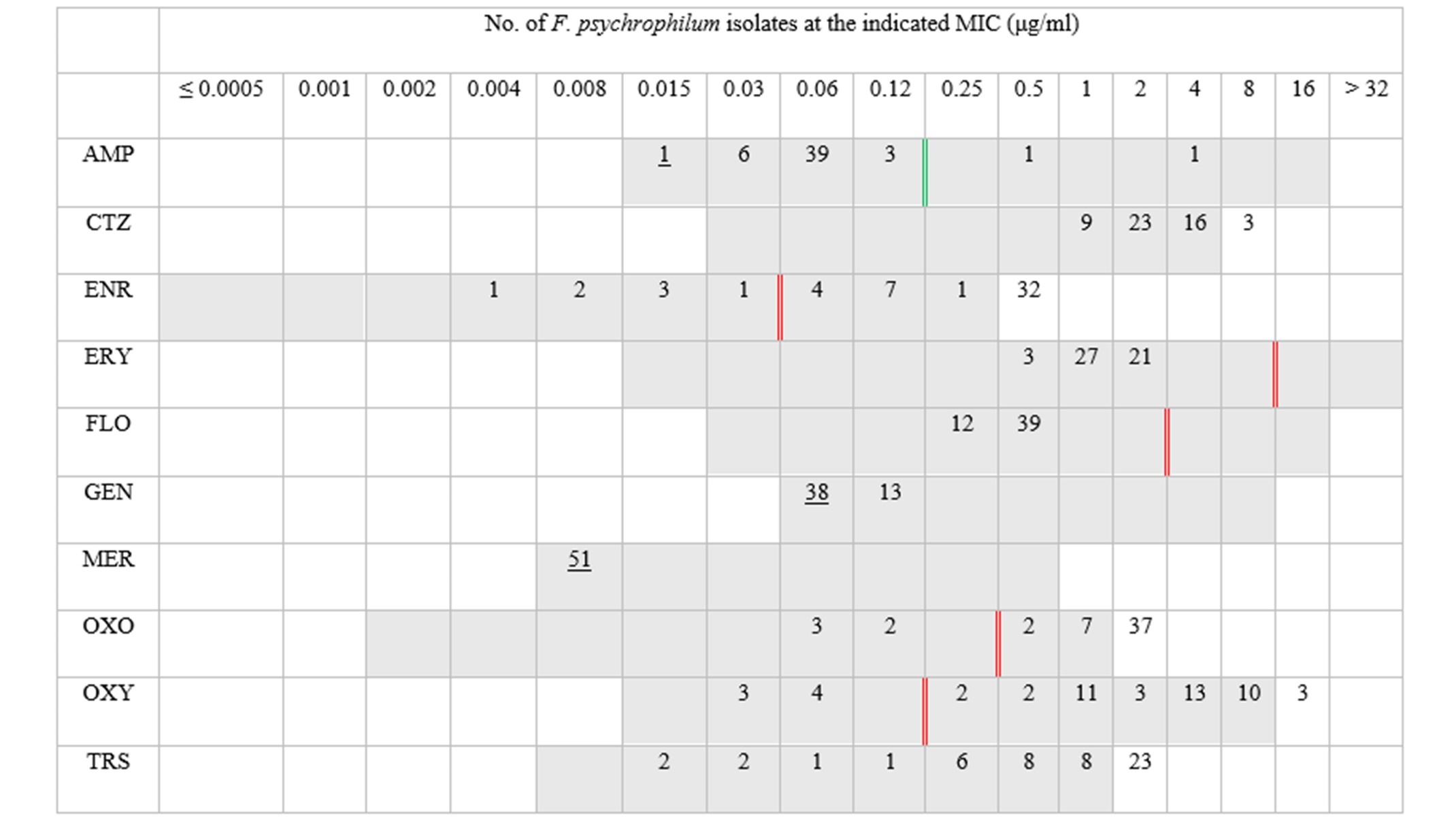


**Legend:** The range of dilutions tested (μg/ml) for each antimicrobial is shaded gray. MICs higher than the highest concentration tested are given as the first concentration above the tested range, and MICs equal to or lower than the lowest concentration tested are underlined. The red and green double lines represent the epidemiological cut-off values (ECVs) and the provisional epidemiological cut-off value for wild type (CO_WT_) for *Flavobacterium psychrophilum*, respectively.

**Figure S1** The distribution of minimum inhibitory concentrations (MICs) of 51 *Flavobacterium psychrophilum* isolates determined using the broth microdilution method for AMP, ampicillin; CTZ, ceftazidime; ENR, enrofloxacin; ERY, erythromycin; FLO, florfenicol; GEN, gentamicin; MER, meropenem; OXO, oxolinic acid; OXY, oxytetracycline; TRS, trimethoprim/sulfamethoxazole.


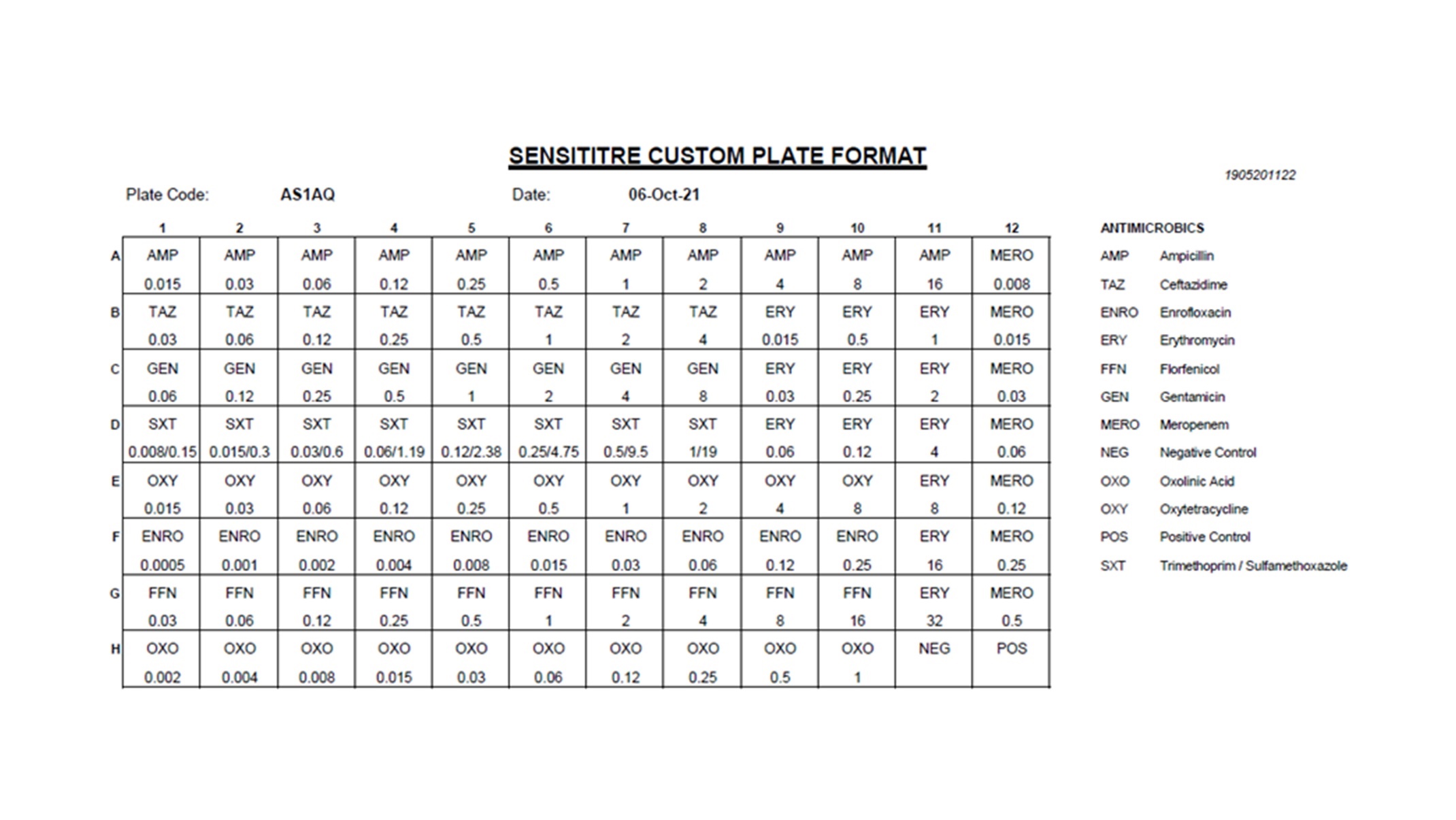


**Figure S2** The layout of the Sensititre custom AS1AQ microtitre plate (Trek Diagnostic Systems).


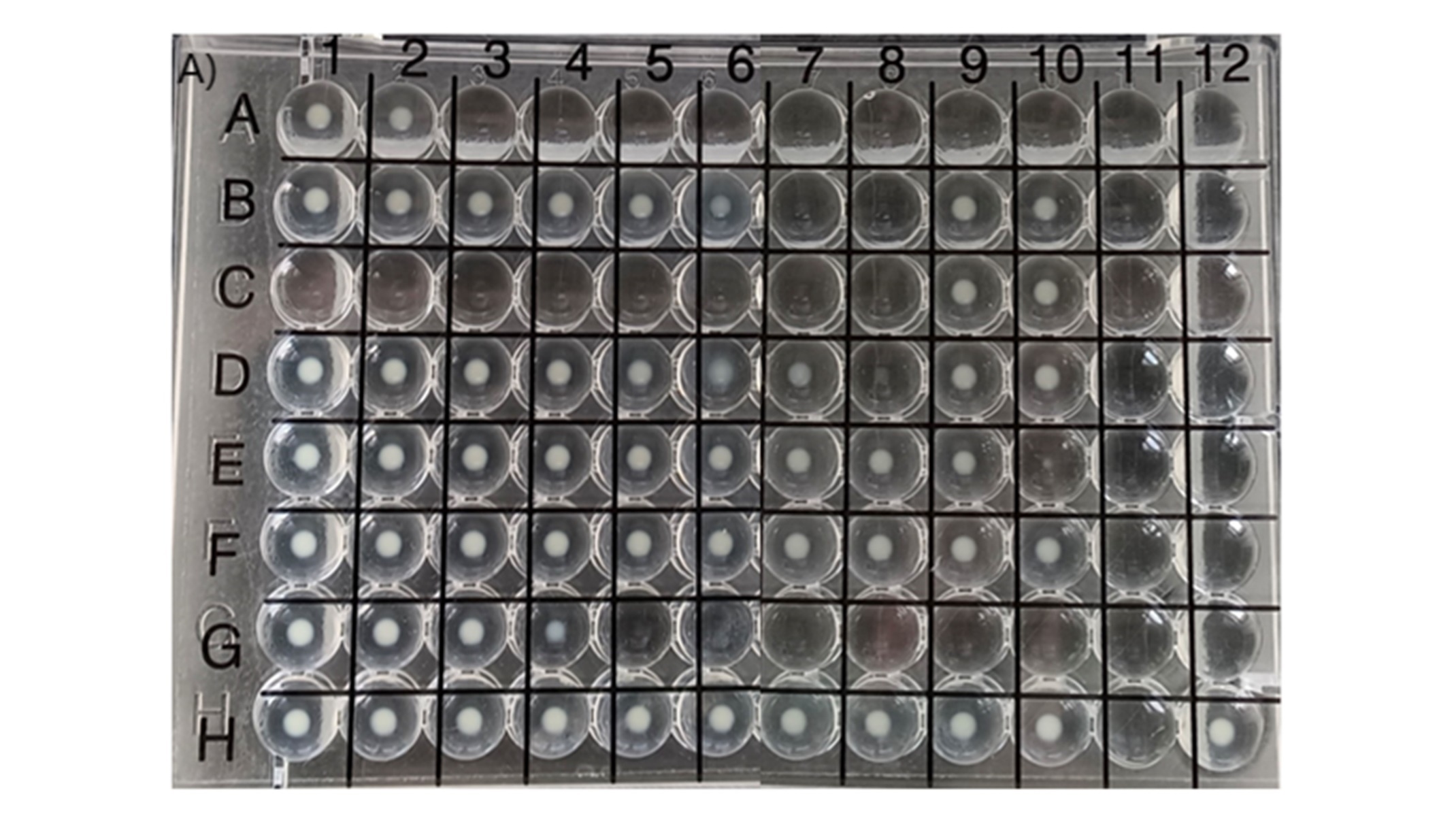


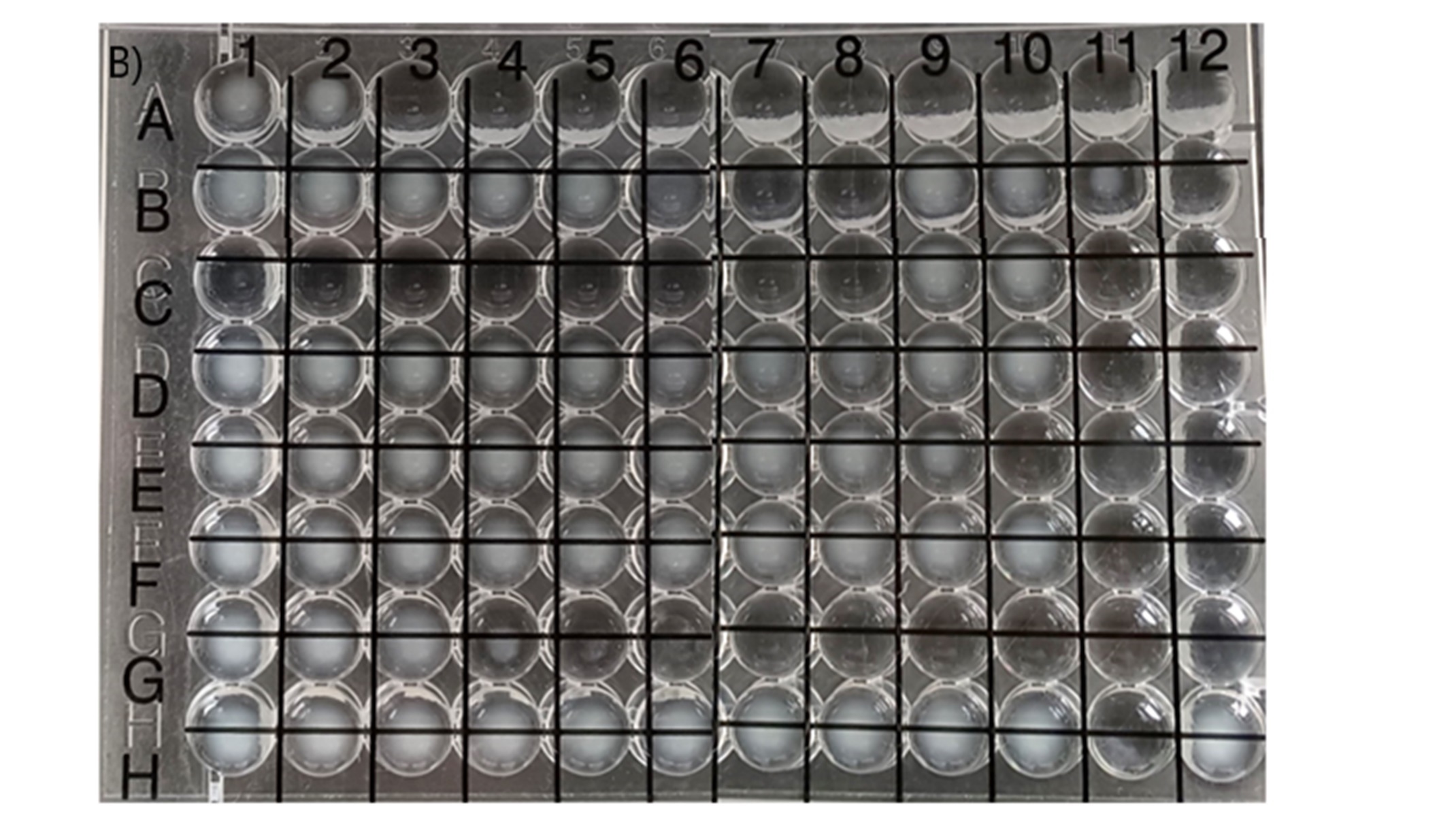


**Figure S****3** Microtitre plates for two selected *Flavobacterium psychrophilum* isolates. A) Isolate no. 47 with smooth edges; B) Isolate no. 17 with rhizoid edges. For each microtitre plate, two images were merged between columns 6 and 7 so that all 96 wells are clearly visible.


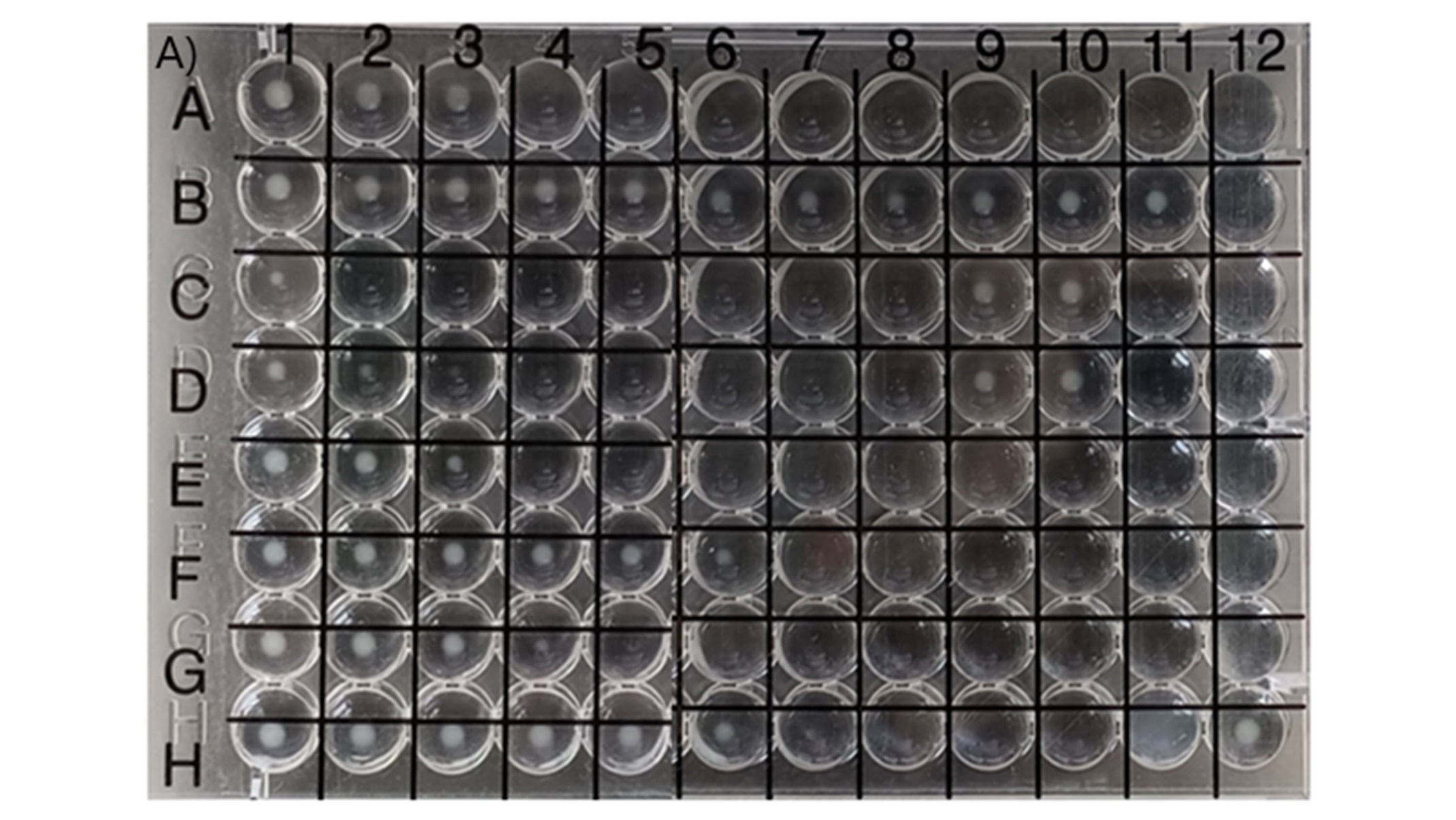


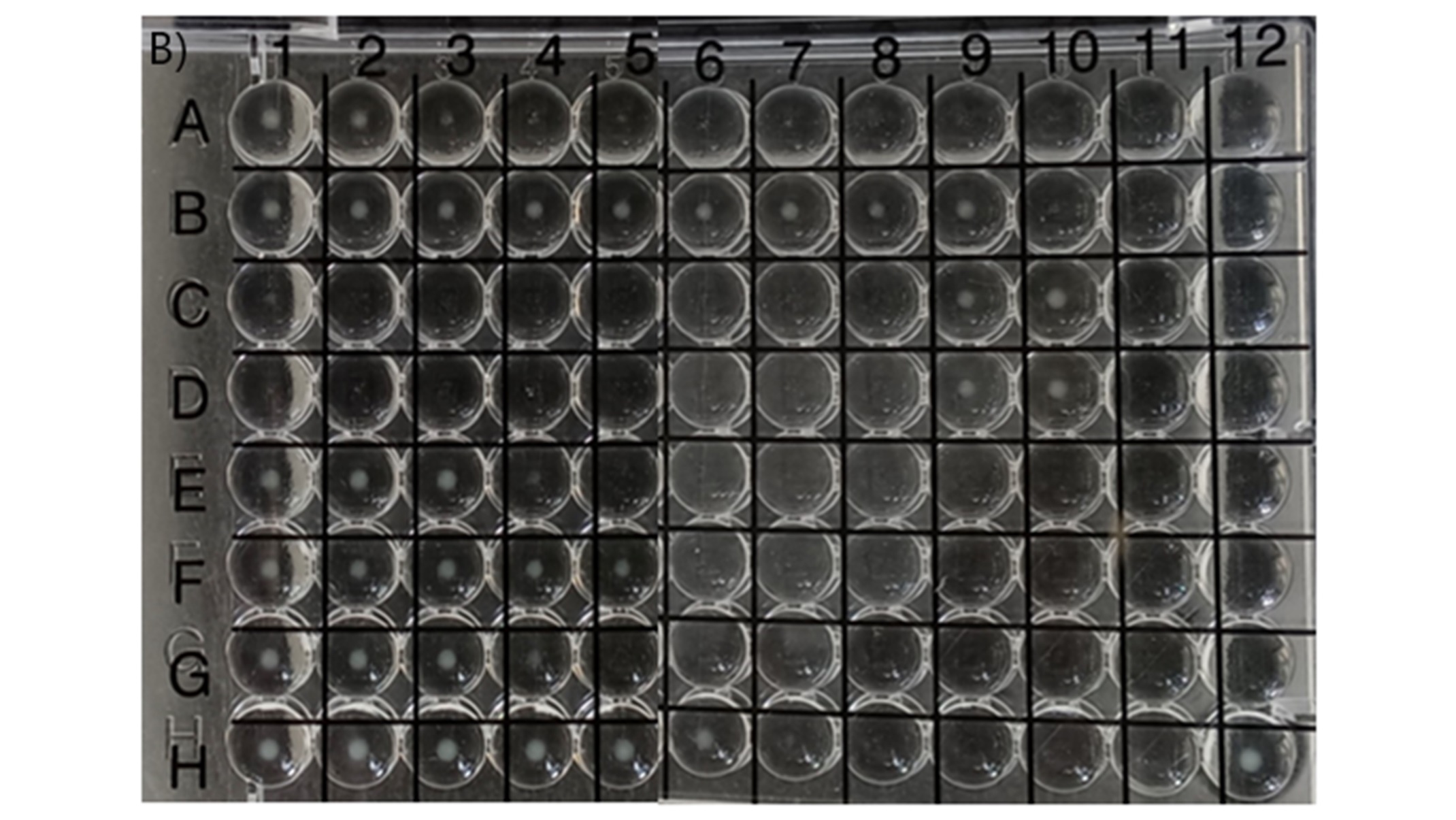


**Figure S4** Microtitre plates for two selected *Flavobacterium branchiophilum* isolates. A) Isolate no. 52 on Anacker and Ordal (AO) broth; B) Isolate no. 53 on diluted cation-adjusted Mueller Hinton broth supplemented with additional cations (DCAMHB+C). For each microtitre plate, two images were merged between columns 5 and 6 so that all 96 wells are clearly visible.
